# TGFβ Governs the Pleiotropic Activity of NDRG1 in Triple-Negative Breast Cancer Progression

**DOI:** 10.7150/ijbs.78738

**Published:** 2023-01-01

**Authors:** Araceli López-Tejada, Carmen Griñán-Lisón, Adrián González-González, Francisca E. Cara, Rafael J. Luque, Carmen Rosa-Garrido, José L. Blaya-Cánovas, Alba Navarro-Ocón, María Valenzuela-Torres, Marisa Parra-López, Jesús Calahorra, Isabel Blancas, Juan A. Marchal, Sergio Granados-Principal

**Affiliations:** 1 Department of Biochemistry and Molecular Biology 2, School of Pharmacy, University of Granada, 18011 Granada, Spain.; 2 GENYO, Centre for Genomics and Oncological Research, Pfizer/University of Granada/Andalusian Regional Government, 18016 Granada, Spain.; 3 Instituto de Investigación Biosanitaria ibs.GRANADA, University Hospitals of Granada-University of Granada, Spain; Conocimiento s/n 18100, Granada. Spain.; 4 UGC de Oncología Médica, Hospital Universitario de Jaén, 23007 Jaén, Spain.; 5 UGC de Anatomía Patológica, Hospital Universitario de Jaén, Jaén, Spain.; 6 FIBAO, Hospital Universitario de Jaén, Servicio Andaluz de Salud, Jaén, Spain.; 7 UGC de Oncología, Hospital Universitario “San Cecilio”, 18016 Granada, Spain; 8 Department of Human Anatomy and Embryology, Biopathology and Regenerative Medicine Institute (IBIMER), University of Granada, 18011 Granada, Spain.; 9 Excellence Research Unit “Modeling Nature” (MNat), University of Granada, Spain.

**Keywords:** Cancer stem cell, NDRG1, TGFβ, Triple-negative breast cancer, Tumor progression

## Abstract

In triple-negative breast cancer (TNBC), the pleiotropic NDRG1 (N-Myc downstream regulated gene 1) promotes progression and worse survival, yet contradictory results were documented, and the mechanisms remain unknown. Phosphorylation and localization could drive NDRG1 pleiotropy, nonetheless, their role in TNBC progression and clinical outcome was not investigated. We found enhanced p-NDRG1 (Thr346) by TGFβ1 and explored whether it drives NDRG1 pleiotropy and TNBC progression. In tissue microarrays of 81 TNBC patients, we identified that staining and localization of NDRG1 and p-NDRG1 (Thr346) are biomarkers and risk factors associated with shorter overall survival. We found that TGFβ1 leads NDRG1, downstream of GSK3β, and upstream of NF-κB, to differentially regulate migration, invasion, epithelial-mesenchymal transition, tumor initiation, and maintenance of different populations of cancer stem cells (CSCs), depending on the progression stage of tumor cells, and the combination of TGFβ and GSK3β inhibitors impaired CSCs. The present study revealed the striking importance to assess both total NDRG1 and p-NDRG1 (Thr346) positiveness and subcellular localization to evaluate patient prognosis and their stratification. NDRG1 pleiotropy is driven by TGFβ to differentially promote metastasis and/or maintenance of CSCs at different stages of tumor progression, which could be abrogated by the inhibition of TGFβ and GSK3β.

## Introduction

Triple-negative breast cancer (TNBC) is an aggressive breast cancer subtype representing approximately 10 to 15% of all new cases diagnosed with breast cancer. It is characterized by the absence of estrogen, progesterone, and HER2 receptors, which influences the lack of targeted therapies for its treatment. TNBC displays poor survival rates due to a high risk of metastasis and relapse, drug resistance, invasiveness, tumor cell proliferation, and heterogeneity 1,2. Hence, the identification of new predictive and prognostic biomarkers will allow alternative patient stratifications and the development of novel targeted therapies to enhance patient survival.

The role of N-myc downstream-regulated gene 1 (NDRG1) in human cancer is controversial and remains unclear. NDRG1 exhibits a pleiotropic activity depending on the types of tumors and tissues as several studies identified NDRG1 as a tumor and metastasis suppressor in colorectal, prostate, breast, or pancreatic cancer 3-5, but it showed a pro-oncogenic role in other cancers, including aggressive breast cancer, by promoting tumor growth, metastasis, angiogenesis and poor prognosis 6-9. Multiple phosphorylation sites have been described for this protein, and the relationship between post-translational modifications, function, regulation, and subcellular localization of NDRG1 has been established. Indeed, NDRG1 phosphorylation at Thr346 and Ser330 is a common factor present in multiple cancer types, as well as their localization in cytoplasm and nucleus, respectively 8,10,11. Even if it has been suggested that NDRG1 subcellular localization, phosphorylation, and interaction with other molecules could be responsible for such a pleiotropy, the underlying reasons remain elusive 8. Subcellular localization of NDRG1 exemplifies its cancer-type-dependent pleiotropy, for instance, nuclear NDRG1 protein expression is associated with poor prognosis in colorectal cancer 12 and favorable prognosis in renal carcinoma 13. This duality was also seen in breast cancer, with NDRG1 being reported as a metastasis suppressor and tumor promoter in estrogen-receptor (ER) positive and negative breast cancers, respectively 9,14-18. In TNBC, NDRG1 expression was found to correlate with poorer patient survival, however, inconsistent experimental results across the studies within the same tumor subtype indicate not only a cell-dependent pleiotropy but also that the underlying mechanisms that trigger NDRG1 pleiotropy are still unknown 9,17,18. Importantly, as none of the studies on breast cancer have evaluated the association between subcellular localization of total and phosphorylated NDRG1 and patient prognosis, in this study we aimed to investigate the correlation between those two important players of NDRG1 pleiotropy and patient survival and to give an answer to the experimental inconsistencies previously reported.

It has been proposed that the pleiotropic effects of NDRG1 are also exhibited by having a different role on the same molecules in distinct cancers and cell types, such as β-catenin 8. In this regard, NDRG1 inhibited TGFβ (transforming growth factor β)-induced Wnt/β-catenin signaling and epithelial-mesenchymal transition (EMT) in colon and prostate cancer cells 19. Interestingly, in our previous investigations, we found an increased expression of NDRG1 phosphorylated at Thr346 in TNBC cell lines stimulated with TGFβ1 20, suggesting that NDRG1 could have a pro-oncogenic function downstream TGFβ signaling. Given that TGFβ is a well-known pleiotropic molecule 21, we also investigated in the present study whether TGFβ could be one mechanism that dictates NDRG1 pleiotropy.

## Materials and Methods

### Analysis of breast cancer patient's survival in public datasets

The association of *NDRG1* mRNA expression with relapse-free survival was analyzed with KM plotter (https://kmplot.com/analysis/) and GOBO (Gene expression-based Outcome for Breast cancer Online) 22,23 in all cases of breast cancer, luminal A, luminal B, HER2^+^, basal-like, and TNBC. In the KM plotter, patients were split by median expression of *NDRG1* and by three quantiles (low, median, high expression) in GOBO. Multivariate analysis of *NDRG1* expression groups by GOBO was made with the covariates tumor size and histological grade, and ER and lymph node (LN) status. In both databases, the Affymetrix probe 200632_s_at was used for the analysis.

### Immunohistochemistry

Sections (4 µm) of formalin-fixed, paraffin-embedded tumor biopsies of TNBC, included in tissue microarrays (TMAs) provided by the Andalusian Health Service Biobank, were treated for deparaffinization, rehydration, and antigen retrieval using standard procedures (EnVision FLEX reagents, Agilent Dako). Before immunohistochemistry, antigen retrieval was performed at pH 6.0, for 20 min at 97 °C, according to the manufacturer's instructions. After washing in diluted EnVision FLEX Wash Buffer, slides were incubated in EnVision FLEX Peroxidase-Blocking Reagent solution for 5 min, then washed and stained at room temperature on an automated system (Autostainer link 48, Dako) with primary antibodies against NDRG1 (D8G9), phospho-(p)-NDRG1(D98G11) (both from Cell Signaling; 1:75 dilution), p-GSK3β (Tyr216) (Novus Biologicals; 1:50 dilution) and TGFβ1 (Elabscience; 1:50 dilution) for 20 min, washed, incubated 20 min with Dako EnVision FLEX/HRP), washed, incubated 8 min with EnVision FLEX Substrate Working solution and counter-stained with hematoxylin (EnVision FLEX Hematoxylin). Sections were then dehydrated and mounted in a Leica CV5030 automatic mounter with mounting media (Panreac). Staining intensity was assessed by a pathologist as 0 (negative), 1+ (weak), 2+ (moderate) and 3+ (intense). For NDRG1 and p-NDRG1 evaluation, a score was also established by multiplying the intensity and % of stained cells, therefore, the score ranged from 0 to 300. Intensity and extension of the staining were performed on digital images obtained with a 3DHISTECH preparation scanner.

### Cell culture

Primary-tumor-derived (BT549, Hs578T) and pleural-effusion-derived cell lines (MDA-MB-231, MDA-MB-436, MDA-MB-468) were purchased from ATCC. The primary tumor-derived cell line SUM159PT was obtained from Asterand Bioscience. All cell lines were maintained in DMEM medium (Sigma-Aldrich) supplemented with 10% FBS (Thermo Fisher Scientific) and 1% antibiotic-antimycotic (Gibco) (growth medium). Cells were maintained at 37 °C and 5% atmospheric CO_2_.

### Immunofluorescence analysis

Cells were fixed with 4% paraformaldehyde in PBS for 20 min at room temperature (RT), blocked for 1h at RT in PBS with an Immunofluorescence Application Solutions Kit (Cell Signaling), and incubated with the primary antibody overnight at 4 °C. Primary antibodies were NDRG1 (D8G9) (1:200 dilution) and p-NDRG1 (Thr346) (D98G11) (1:800 dilution) (Cell Signaling). Further, samples were washed three times with PBS, incubated with the secondary antibody (Alexa Fluor 488) (1:500 dilution) for 1h at RT, washed three times with PBS, and mounted with Prolong Gold antifade reagent with DAPI (Cell Signaling). Images were acquired with a Zeiss LSM 710 confocal microscope.

### Gene knockdown, TGFβ stimulation, and treatment with pharmacological inhibitors

*NDRG1* or *GSK3B* genes were inhibited by transient transfection with siRNA (siNDRG1 and siGSK3β) from Santa Cruz (50 ng/ml) and lipofectamine RNAiMAX (Invitrogen) following the manufacturer's instructions for 48h after stimulation with TGFβ1 (10 ng/ml) (PeproTech) for 8h or 14 days. TGFβ1 was replenished every 72h. Scrambled siRNA (SCR) was used as negative control 20. Galunisertib (LY2157299, TGFβR1 inhibitor) (5 μM), CHIR99021 (GSK3α/β inhibitor) (10 μM), LY294002 (PI3K inhibitor) (10 μM), MK2206 (Akt inhibitor) (1 μM), GSK650394 (SGK1/2 inhibitor) (10 μM), and Rapamycin (mTOR inhibitor) (10 μM) were from MedChemExpress. Cells were pre-stimulated with TGFβ1 for 8h and treated with each inhibitor for 24, 48, and 72h.

### Wound healing and invasion assays

Wound healing was made with cells cultured in 6-well plates until 80% confluence. Then, a wound was made in the cell monolayer with a 100 µl-pipette tip. After washing with PBS, fresh growth medium was added. Photos were taken at 0, 14, and 24h after the wound was made. Cell migration was analyzed with ImageJ. Tumor cell invasion was determined with the Cultrex BME Cell Invasion Assay kit (Trevigen) as we published 20.

### Western blotting

Cells were collected and lysed in 1X lysis buffer (Cell Signaling) and 1X protease/phosphatase inhibitor cocktail (Thermo Scientific). Samples (30 μg protein) were boiled in sample buffer (Thermo Fisher Scientific) containing β-mercaptoethanol (Sigma-Aldrich), separated by SDS-PAGE electrophoresis in polyacrylamide gels, and transferred onto nitrocellulose membranes (Bio-Rad). Membranes were incubated overnight at 4 °C with the primary antibody (1:1000 dilution), and the secondary antibody for 1 hour (1:2000 dilution). Protein bands were detected with the ImageQuantLAS4000 digital imager. Antibodies against Vimentin (D21H3), Snail (C15D3), Slug (C19G7), p-NDRG1 (Thr346, D98G11), NDRG1 (D8G9), p-p65 (Ser536, 93H1), p65 (D14E12) and β-Actin (13E5) were from Cell Signaling; GSK3β, p-GSK3β (Tyr216) and p-GSK3β (Ser9) from Novus Biologicals; Twist (Twist2C1a) and MDR1 (D-11) from Santa Cruz; GAPDH (1E6D9) was from Proteintech.

### Mammosphere culture

Mammospheres were cultured with a patented culture media (WO2016020572A1) 24 which contains DMEM/F-12 supplemented with 1X B-27 (Invitrogen), 4 ng/ml heparin (Sigma-Aldrich), 10 µg/ml Insulin-Transferrin-Selenium (Invitrogen), 1 mg/ml hydrocortisone (Sigma-Aldrich), 10 ng/ml epidermal growth factor (Sigma-Aldrich), 10 ng/ml fibroblast growth factor (Sigma-Aldrich), 10 ng/ml interleukin 6 (Miltenyi), and 10 ng/ml hepatocellular growth factor (Miltenyi). Cells treated with TGFβ1 and transfected with siNDRG1 or SCR were cultured in ultra-low attachment plates to form primary mammospheres (1MS). To minimize siRNA leakage, a second transfection (25 nM siRNA) was performed at the time of seeding upon mammosphere-forming conditions. Mammospheres were manually counted after 72h, trypsinized, and cultured without additional treatment for 72h to form secondary mammospheres (2MS). Mammosphere-forming efficiency (MSFE) was calculated by counting the number of mammospheres with a diameter >50µm. *NDRG1* inhibition in CSC-enriched mammosphere cultures 24 was performed with siNDRG1, SCR, and TGFβ1 in 2MS of SUM159 and MDA-MB-231 cells, which were derived from 1MS without stimulation and transfection, and MSFE was evaluated in the third generation (3MS). To determine the effect of LY2157299 (LY) and/or CHIR99021 (CHIR), cells were treated with drugs (replenished at 48h) and TGFβ1 when seeded in mammosphere media. 1MS were counted at day 4, trypsinized, and cultured without drugs to form 2MS for 4 (SUM159) or 7 days (MDA-MB-231 and BT549).

### Soft-agar colony formation

Transfected and TGFβ1-stimulated cells were seeded in a 0.8% low-melting-point agarose layer on top of a 1.6% low-melting-point agarose layer in 6-well culture plates. To minimize siRNA leakage, a second transfection (25 nM siRNA) was performed at the time of seeding upon colony-forming conditions. Then, the cells were incubated for 21 days (SUM159 for 14 days) at 37 °C and 5% CO_2_. Colony formation (diameter >50 µm) was examined under a microscope after staining with 0.05% crystal violet (Sigma-Aldrich).

### Flow cytometry

Aldehyde dehydrogenase 1 (ALDH1) enzyme activity was assayed with the Aldefluor assay kit (StemCell Technologies) following the manufacturer's instructions. TGFβ1-stimulated and transfected cells were incubated with the Aldefluor reagent for 45 min at 37 °C or DEAB (diethylaminobenzaldehyde) as the negative control. CD44⁺/CD24^-/low^ cell populations were also evaluated by staining with anti-CD44-PE and anti-CD24-FITC (BD Pharmingen) or their isotype controls at 4 °C for 15 min. The effect of LY and/or CHIR was assessed in 2MS after treatment with the inhibitors and TGFβ1 for 72h. ALDH1^+^ and CD44⁺/CD24^-/low^ subpopulations were analyzed in a FACSVerse (BD Biosciences) flow cytometer. The side population was determined by Hoechst 33342 dye exclusion assay. Briefly, after stimulation with TGFβ1 and transfection with siRNA, cells were incubated for 90 min at 37 °C in DMEM supplemented with 2% FBS, 10 mM HEPES, and 5 µg/ml Hoechst 33342 (Sigma-Aldrich) in the dark with interval mixing. Verapamil (50 µM) was used as a control of inhibition. Hoechst 33342 was excited with a UV laser at 355 nm, and emissions were detected at 450/50 nm (Hoechst blue) and 670/30 nm (Hoechst red) in a FACSAria III cell sorter (BD Biosciences).

### Cell proliferation

Proliferation was measured in SUM159 and MDA-MB-231 cells treated with TGFβ1 (10 ng/ml) and the pharmacological inhibitors LY (5 μM) and/or CHIR (10 μM) by WST-1 (Merk) at 48 and 72h, respectively, at 450 nm using an M200 Nanoquant plate reader (Tecan).

### Statistical analysis

In patient samples, numeric variables are described as mean and standard deviation, and categorical variables as frequency and percentage. Normality study of numeric variables was done with the Shapiro-Wilk test. Spearman correlation was performed to find correlations between different markers. Patient survival analysis was calculated through Kaplan-Meier and Log-rank test. To determine what variables were risk or protector factors at decease, individual Cox regression, raw Hazard Ratio (HR), and Confidence Interval (CI) were calculated. For numeric variables, a threshold was calculated according to Youden's index with receiver operating characteristic (ROC) curves 25. For the Cox multivariate regression model, variables with a p<0.20 were included, and adjusted HR and CI were calculated. For all analyses, p<0.05 was statistically significant. Statistical analyses were run with IBM SPSS v21.0 software. All *in vitro* data were analyzed using GraphPad Prism software. Data are presented as mean ± SEM. Differences between the two groups were analyzed by a two-tailed Student's t-test. A p-value <0.05 was considered significant.

## Results

### High expression of NDRG1 protein is correlated with poorer survival of TNBC patients

**Figure 1:**
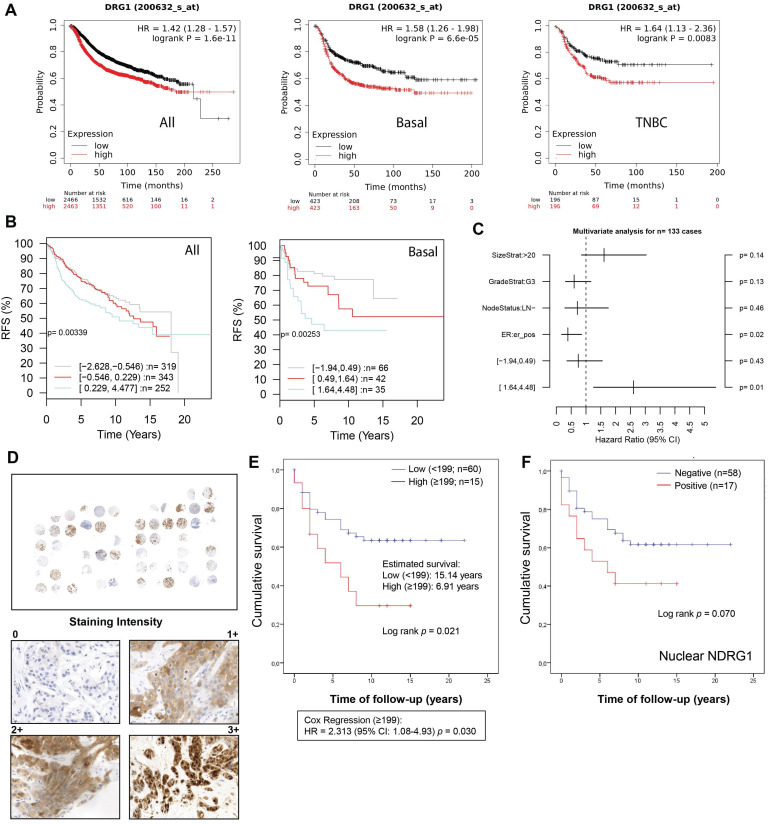
** NDRG1 expression correlates with poor patient survival in TNBC patients. A** Kaplan-Meier curve by the KM plotter database shows that high *NDRG1* expression correlates with poorer relapse-free survival (RFS) in all cases of breast cancer, luminal B, basal-like, and TNBC. **B** Kaplan-Meier analysis by GOBO online tool shows that higher *NDRG1* gene expression is correlated with poorer RFS in all cases of breast cancer and basal subtype. **C** Multivariate analysis with GOBO of *NDRG1* gene expression in breast cancer (n=133). **D** Representative images of negative, 1+, 2+, and 3+ NDRG1 staining intensity in tumor tissue of TNBC patients (original optical objective: 40×). **E** Kaplan-Meier shows that NDRG1 protein high expression is associated with poorer cumulative patient survival in tumor tissue of TNBC patients (n= 75). **F** Kaplan-Meier analysis by positive vs. negative nuclear NDRG1 staining in tumor tissue of TNBC patients (n= 75).

In public datasets, *NDRG1* gene expression has been found higher in the aggressive breast cancer subtypes basal-like, HER2^+^, and TNBC, which was correlated with poorer overall survival 9,17,18,26. Accordingly, in the KM plotter database, we found that high* NDRG1* expression correlated with less relapse-free survival (RFS) in all cases of breast cancer (HR: 1.42; p= 1.6×10^-11^), luminal B (HR: 1.27; p=0.008), basal-like (according to StGallen guidelines) (HR: 1.58; p=6.6×10^-5^) and TNBC (ER^-^, PR^-^, HER2^-^) (HR: 1.64; p=0.008) (Fig. 1A; Fig. S1A). On the contrary, no correlation was observed between high NDRG1 expression and RFS in Luminal A and HER2^+^ molecular subtypes (StGallen guidelines) (Fig. S1A). Similarly, gene sets analysis by GOBO online tool showed that the higher *NDRG1* gene expression, the poorer relapse-free survival in all cases of breast cancer (p=0.003) and basal subtype (p=0.002) (Fig. 1B). However, such a correlation was not observed in Luminal A, Luminal B, and HER2^+^ breast cancer subtypes (Fig. S1B). A multivariate analysis with GOBO indicated that the higher *NDRG1* gene expression is significantly associated with a higher hazard ratio (HR: ~2.5; p=0.01) in basal-like breast cancer patients than a lower expression (HR: ~0.5; p=0.43) (Fig. 1C).

To validate these observations, we analyzed NDRG1 expression by immunohistochemistry in tumor tissue (n=75) collected from a total cohort of 83 TNBC patients after surgery (Table 1). Age, tumor size, vascular invasion, and treatment regimen were significantly correlated with patient survival. Specifically, an age ≥73 years (ROC analysis according to the Youden index) showed a mean survival of 6.92 years *versus* <73 years (p=0.037), T3-T4 (1.8 years) *versus* T1 and T2 (p=0.003) and positive vascular invasion (2.66 years) *versus* negative (p=0.015). Additionally, in agreement with previous reports 27, in our cohort, those patients who received adjuvant (n=51, 64.6%) showed longer survival (~16.7 years) compared to neoadjuvant therapy (n=17, 21.5%) (~6 years) (p=0.004) (Table 1). In our patient cohort, NDRG1 protein expression was positive in 56 patients (~75%), being mostly cytoplasmic (~67%), but also membrane (33%) and nuclear (~27%) (Table 2), as previously reported 9,26. Interestingly, membrane and cytoplasmic staining (1+ and 2+ intensity) did not show nuclear staining, however, the highest staining intensity (3+) showed both nuclear and cytoplasmic distribution of NDRG1 (Fig. 1D). We next sought to analyze whether NDRG1 protein expression was associated with patient survival. Our results demonstrated that a high expression of NDRG1 (score ≥199) was associated with poorer cumulative patient survival (mean survival: 6.9 years) compared to a lower expression (score <199) (mean survival: 15.1 years) (p=0.021) (HR = 2.313; 95% CI: 1.08-4.93; p=0.030) (Fig. 1E). Although not significant, higher NDRG1 nuclear staining was also associated with less cumulative patient survival (p=0.070), and no differences were found for cytoplasmic and membrane staining (Fig. S1C). Interestingly, when cases were analyzed by positive *vs.* negative NDRG1 staining, only those with positive nuclear expression showed less survival (9.4 years) than negative staining (16.6 years), but it was not significant (p=0.070) (Fig. 1F and Table 2).

### Cellular expression and subcellular localization of NDRG1 and p-NDRG1 (Thr346) in TNBC tumor tissue correlate with patient survival and TGFβ1 expression

**Figure 2:**
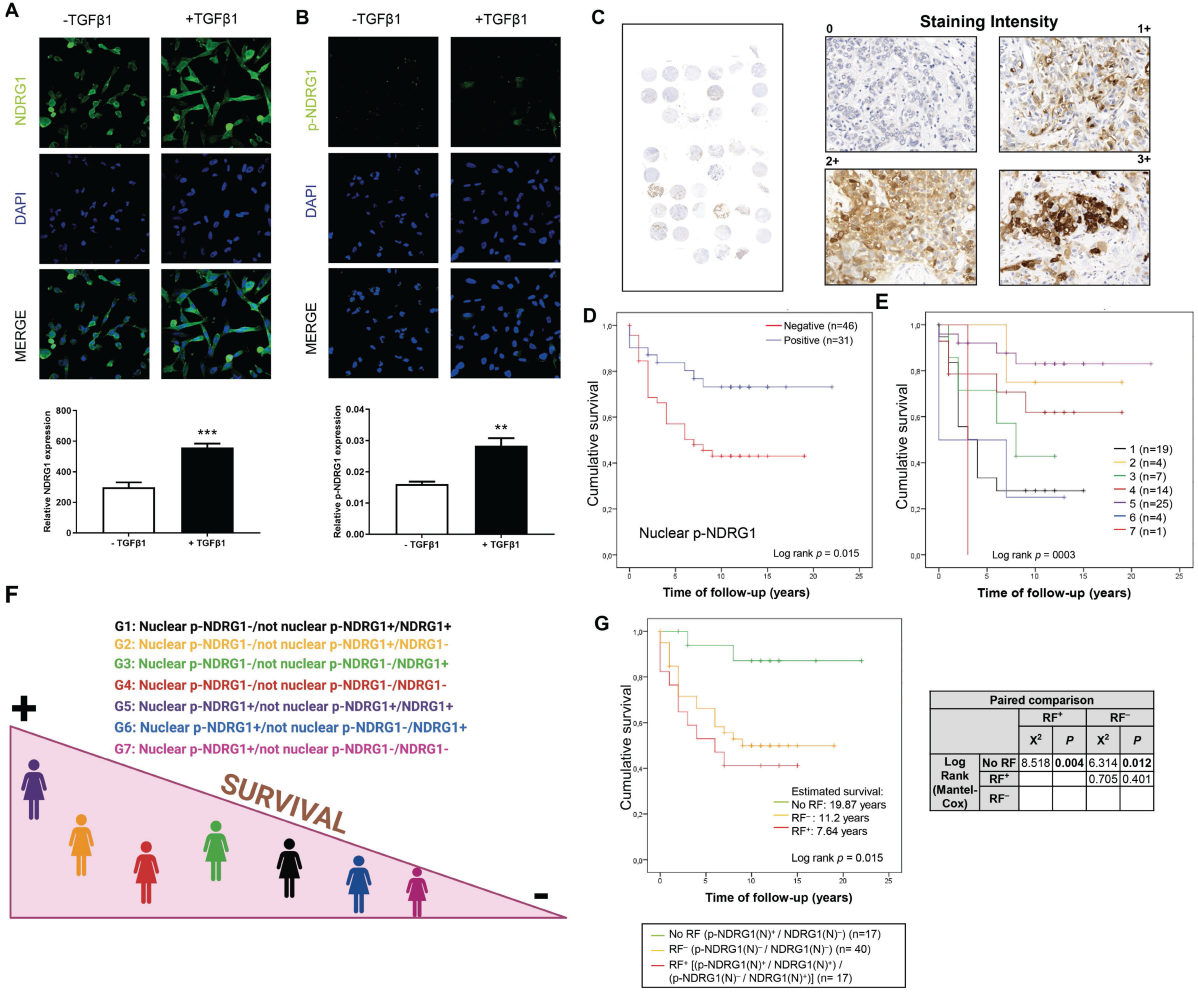
** Cellular expression and subcellular localization of NDRG1 and p-NDRG1 (Thr346) in TNBC correlates with patient survival and TGFβ1 expression. A** Representative confocal images and quantification of the fluorescence intensities of NDRG1 and **B** p-NDRG1 (Thr346) in MDA-MB-231 cells treated or not with TGFβ1. The average fluorescence intensities were calculated from three parallel immunofluorescence images. Original magnification: 40×. **C** Representative images of negative, 1+, 2+, and 3+ p-NDRG1 (Thr346) staining intensity in tumor tissue of patients with TNBC (original optical objective: 40×). **D** Kaplan-Meier analysis of cumulative survival of nuclear p-NDRG1 staining in tumor tissue of TNBC patients (n= 77). **E** Analysis of cumulative survival after diagnosis of TNBC patients stratified by differential staining and subcellular localization of global NDRG1 and p-NDRG1 (n= 74). 1: Nuclear p-NDRG1^-^/not nuclear p-NDRG1^+^/NDRG1^+^ (n=19); 2: Nuclear p-NDRG1^-^/not nuclear p-NDRG1^+^/NDRG1^-^ (n=4); 3: Nuclear p-NDRG1^-^/not nuclear p-NDRG1^-^/NDRG1^+^ (n=7); 4: Nuclear p-NDRG1^-^/not nuclear p-NDRG1^-^/NDRG1^-^ (n=14); 5: Nuclear p-NDRG1^+^/not nuclear p-NDRG1^+^/NDRG1^+^ (n=25); 6: Nuclear p-NDRG1^+^/not nuclear p-NDRG1^-^/NDRG1^+^ (n=4); 7: Nuclear p-NDRG1^+^/not nuclear p-NDRG1^-^/NDRG1^-^ (n=1). **F** Illustration that summarizes how stratifications groups are differentially associated with patient survival. Created with BioRender.com. **G** Impact of nuclear NDRG1 and p-NDRG1 staining status as risk factors of shorter cumulative survival after diagnosis of TNBC patients. No Risk Factor (RF) (p-NDRG1(N)^+^/NDRG1(N)^-^), RF^-^ (p-NDRG1(N)^-^/NDRG1(N)^-^) and RF^+^ [(p-NDRG1(N)^+^/NDRG1(N)^+^) / (p-NDRG1(N)^-^/NDRG1(N)^+^)] and paired comparison by Chi-square test (n=74).

We previously reported an enhanced expression of p-NDRG1 (Thr346) in TNBC cell lines upon stimulation with TGFβ1 20. Phosphorylation of NDRG1 at Thr346 (among other sites) was proposed as pro-oncogenic in certain cell types where NDRG1 promotes tumor progression 11, and it has been associated with oncogenic markers such as mTORC2 activation 20,28 and PTEN depletion 11. On the other side, p-NDRG1 (both at Thr346 and Ser330) was associated with tumor suppressor activity through the inhibition of NF-κB 29. To clarify these contradictory results, we studied if NDRG1 and/or p-NDRG1 (Thr346) could be involved in tumor aggressiveness mediated by TGFβ1. Our previous data were validated herein in two TNBC cell lines (SUM159 and MDA-MB-231) treated with a single stimulation of TGFβ1. We found an increased expression of p-NDRG1 (Thr346) at 8h that was maintained during 48h and started to decrease at 72h (Fig. S2A). Given that it was initially suggested that the subcellular location of NDRG1, p-NDRG1 (Ser330, Thr346) could determine its pleiotropic effect in tumor cells 11, we questioned if TGFβ1 drives changes in the subcellular localization of NDRG1 and p-NDRG1 (Thr346). Confocal imaging in MDA-MB-231 cells showed that high levels of NDRG1 were localized both in the cytoplasm and nucleus, and 100% of cells showed positive staining. On the contrary, a very low expression of p-NDRG1 (Thr346) was found in a few cells and predominantly localized in the cytoplasm (Fig. 2A and B; Fig. S2B), as previously described 11. Stimulation with TGFβ1 enhanced the expression of NDRG1 and p-NDRG1, as well as the number of positive cells of the latter, but it did not entail alterations in their subcellular localization (Fig. 2A and B; Fig. S2B). Our results suggest that nuclear expression is not a crucial factor of NDRG1 pleiotropy, which supports the hypothesis of previous studies 8. We next questioned whether p-NDRG1 (Thr346) staining status and its subcellular localization could have an impact on TNBC patient survival. Accordingly, we further investigated the protein expression of p-NDRG1 (Thr346) in TNBC tumor tissue (Fig. 2C) and whether it was associated with patient survival. We found that 56 (out of 77) cases were positive for p-NDRG1 staining (~73%) with no evidence of association with patient survival (Table 2). We next analyzed the subcellular localization of p-NDRG1 and found that most of the cases (n=51; ~66%) expressed it in the cytoplasm, which was not significantly correlated with patient survival. Membrane expression was negative in 74 cases (~96%). In contrast, we found negative nuclear expression in several cases (n=46; ~60%) that was associated with less patient mean survival (10 years) compared to positive staining (17 years) (p=0.015) that showed protective effects (Fig. 2D). This association was validated through a univariate Cox regression (HR= 2.52; p=0.023) (Table 2). Similar results were obtained when a staining score was implemented and found that the very low score (<7.5 out of 300 as maximum) for nuclear p-NDRG1 was associated with poorer patient survival *vs.* a score of ≥7.5 (Fig. S2C). On the contrary, the cytoplasmic p-NDRG1 score threshold was set at 12.5, with no association with patient survival (Table S1). Taken together, these results confirmed that p-NDRG1 expression is predominantly cytoplasmic, as reported by others 9,11,26. Notably, we herein showed for the first time that loss of p-NDRG1 nuclear expression can be a common event in TNBC that could negatively affect patient survival. Nevertheless, it will be necessary to decipher how the latter might be modulated by other cellular locations of p-NDRG1 and total NDRG1 staining status (see patient stratification below). Overall, our data suggest that both the positiveness status and subcellular localization of NDRG1 and p-NDRG1 (Thr346) are relevant for the survival of patients with TNBC.

NDRG1 is known to have both oncogenic and anti-tumor/anti-metastasis roles depending on the tumor type 30, but the reasons are still unknown. Because we found that NDRG1 and its phosphorylation at Thr346 were altered by TGFβ1, we next tested whether their expression levels were correlated in TNBC patient tumor tissue. TGFβ1 staining was positive in 57 cases out of 69 (82.6%) and it was mostly localized in the cytoplasm of tumor cells (83.8%) (Fig. S2D; Table S2), as previously reported 31. Spearman's rank test demonstrated that TGFβ1 expression was positively correlated with nuclear NDRG1 (NDRG1(N)). According to subcellular expression, membrane TGFβ1 was correlated with both NDRG1(N) and cytoplasmic p-NDRG1 (pNDRG1(C)), whereas there was a correlation between cytoplasmic TGFβ1, total NDRG1 and NDRG1(N). Finally, we found a correlation between membrane TGFβ1 and cytoplasmic NDRG1 (NDRG1(C)) and p-NDRG1(C) (Table 3). TGFβ1 also exhibits a cell-type- and context-dependent pleiotropic effect that promotes or inhibits cancer progression 21; hence, we suggest that this could explain, at least partially, the pleiotropic role of NDRG1. In this sense, it has been recently reported in glioblastoma that GSK3β expression leads to NDRG1 degradation as a tumor suppressor and, conversely, NDRG1 overexpression induced GSK3β degradation as a bidirectional regulatory mechanism 32. Similar to NDRG1 and TGFβ1, GSK3β is known to have a dual antitumor and oncogenic role depending on the tumor type, including breast cancer 33, to overcome chemoresistance in breast cancer 34, and it was recently found to be expressed at high levels in TNBC, what correlated with less patient survival 35. Given that TGFβ1 was found to activate GSK3β (p-GSK3β Tyr216) in MCF10A breast cells and human fibroblasts 36,37, we questioned whether staining of this active form in TNBC tumor tissue (Fig. S2E; Table S2), could correlate with NDRG1 and p-NDRG1 staining. We found that total and cytoplasmic NDRG1 and p-NDRG1 staining positively correlated with total and cytoplasmic p-GSK3β (Tyr216), unlike nuclear p-NDRG1 (p-NDRG1(N)), which positively correlated with total, cytoplasmic and nuclear p-GSK3β (Tyr216) staining. TGFβ1 was reported to promote a transient activation of GSK3β, as p-GSK3β (Tyr216), 36,37, which could explain why we did not find any correlation between TGFβ1 and p-GSK3β staining, and a negative correlation between nuclear TGFβ1 and cytoplasmic p-GSK3β (Table 3).

Overall, our data suggest that NDRG1 pleiotropism could be influenced by its correlation with other pleiotropic proteins such as TGFβ1 and GSK3β, as suggested by other authors 8, and could indicate a potential signaling pathway where NDRG1 is involved in.

### Positive expression of nuclear NDRG1 and negative nuclear p-NDRG1 are risk factors associated with shorter survival after surgery in TNBC patients

Although chemotherapy remains the standard therapeutic approach for TNBC, new treatments are emerging aimed at obtaining meaningful improvements in patient survival 38. The prediction of the risk of distant recurrence and death in response to chemotherapy or other treatment is still a challenge, and histopathological assessment after surgery prevails as one of the most followed ways by clinicians to provide prognostic information 39. In this regard, we aimed to pave the pathway that might give rise to future prediction models for patient prognosis. Therefore, we first questioned whether TNBC patients within our cohort could be stratified according to NDRG1 and/or p-NDRG1 staining and if they could be associated with their survival. Our investigation by Spearman's rank test showed the strongest correlation coefficients between global NDRG1 staining and global p-NDRG1, p-NDRG1(C), and p-NDRG1(N) staining in tumor tissue specimens collected after surgery (Table 3). Accordingly, we stratified patients (n=74) by the combination of global NDRG1 staining status with positive and/or negative nuclear and/or cytoplasmic p-NDRG1 staining, and patient survival was assessed. Because membrane p-NDRG1 staining was positive in only three cases, they were included with p-NDRG1(C) in a category named “not nuclear p-NDRG1” to simplify the stratification criteria. Results of the Kaplan-Meier analysis are depicted in Fig. 2E (p=0.003) and summarized in Fig. 2F. Patients of group 5 (Nuclear p-NDRG1^+^/not nuclear p-NDRG1^+^/NDRG1^+^) showed the highest mean survival time (18,97 years), followed by group 2 (Nuclear p-NDRG1^-^/not nuclear p-NDRG1^+^/NDRG1^-^) (16 years) and group 4 (Nuclear p-NDRG1^-^/not nuclear p-NDRG1^-^/NDRG1^-^) (13.16 years). In contrast, the shortest mean survival was found in group 6 (Nuclear p-NDRG1^+^/not nuclear p-NDRG1^-^/NDRG1^+^) (5 years), followed by group 1 (Nuclear p-NDRG1^-^/not nuclear p-NDRG1^+^/NDRG1^+^) (6 years) and group 3 (Nuclear p-NDRG1^-^/not nuclear p-NDRG1^-^/NDRG1^+^) (7.57 years). Lastly, the only patient in group 7 (Nuclear p-NDRG1^+^/not nuclear p-NDRG1^-^/NDRG1^-^) showed the poorest mean survival (3 years) (Fig. 2E). Pairwise comparison with the Chi-square test of independence showed statistical differences between the patient groups with shorter (1, 3, and 6) and higher survival (group 5). Additionally, the only patient in group 7 showed significantly poorer survival compared with the higher-survival groups 5 and 2 (Table 4).

Based on the association of NDRG1(N) and p-NDRG1(N) with patient survival (Fig. 1F and 2D) as well as our data showing a correlation between TGFβ1, active p-GSK3β, NDRG1, and p-NDRG1, we further questioned whether these markers could be risk factors associated with patient decease. To determine if our markers under study (NDRG1, p-NDRG1, TGFβ1, and p-GSK3β, total and at different subcellular localization) could represent a risk factor associated with higher decease, a multivariate analysis was made by including those markers whose association with patient survival had a p-value <0.20. Only positive NDRG1(N) and negative p-NDRG1(N) were not excluded from the analysis and two models with different possible risk factors were proposed.

Model 1 included the groups No Risk Factor (RF) (p-NDRG1(N)^+^/NDRG1(N)^-^), RF^-^ (p-NDRG1(N)^-^/NDRG1(N)^-^) and RF^+^ [(p-NDRG1(N)^+^/NDRG1(N)^+^) / (p-NDRG1(N)^-^/NDRG1(N)^+^)]. Kaplan-Meier analysis demonstrated that RF^-^ and RF^+^ were associated with poorer patient survival (11.2 and 7.64 years, respectively) compared to No RF (19.87 years) (p=0.015), and it was further validated by a pairwise comparison by the Chi-square test (Fig. 2G). Univariate Cox regression model with clinical variables with p<0.20 from Table 1 (note that vascular invasion was not included due to the low number of positive cases) showed that RF^+^ (HR: 6.97; p=0.012), RF^-^ (HR: 5.07; p=0.029) and age ≥73 years (HR: 2.09; p=0.05), whereas adjuvant therapy and tumor size <3 showed to be protective (Table 5). Multivariate Cox regression model demonstrated that RF^+^ and RF^-^ are risk factors associated with high patient mortality of 18.19-fold (p=0.009) and 8.75-fold (p=0.040), respectively, compared to No RF (Ref. HR:1), those being even higher than an age ≥73 years (HR: 2.09; p=0.05). Again, adjuvant therapy was a protective factor (HR: 0.12; p<0.001) compared to a neoadjuvant approach (Ref. HR: 1) (Table 5).

Similarly, in Model 2 we studied the same biomarker associations but included the possibility of patients showing both p-NDRG1(N)^-^ and NDRG1(N)^+^ combination of staining. Therefore, No RF (p-NDRG1(N)^+^/NDRG1(N)^-^), 1RF^-^ (p-NDRG1(N)^-^/NDRG1(N)^-^), 1RF^+^ (p-NDRG1(N)^+^/NDRG1(N)^+^) and 2RF (p-NDRG1(N)^-^/NDRG1(N)^+^) were analyzed by Kaplan-Meier and found that 1RF^-^, 1RF^+^ and 2RF were correlated with shorter patient survival (11.2, 9.23 and 2.5 years, respectively) *versus* No RF (19.87 years) (p=0002). These results were validated by a paired comparison test (Fig. S2F). In contrast to Model 1, univariate Cox regression model with clinical variables showed that 1RF^+^ (HR: 5.01; p=0.048), 1RF^-^ (HR: 5.10; p=0.028) and 2RF (HR: 18.07; p=0.001), compared to No RF (Table 6). The Multivariate Cox regression model demonstrated that patients with higher mortality were associated with 1RF^+^ (HR: 14.01; p=0.021), 1RF^-^ (HR: 8.49; p=0.043), and 2RF (HR: 30.67; p=0.004) compared to No RF (Ref. HR: 1), those being higher than an age ≥73 years (HR: 4.17; p=0.020). Again, in this model, adjuvant therapy seemed to behave as a protective factor (HR: 0.13; p=0.001) compared to a neoadjuvant approach (Ref. HR: 1) (Table 6). Taken together, because the group 2RF in Model 2 only represents four patients, we herein propose Model 1 to describe two novel risk factors that are highly associated with the mortality of the patients within our cohort.

### NDRG1 pleiotropic activity on EMT, metastasis, and tumor-initiating abilities depends on the origin of tumor cells and TGFβ stimulation

**Figure 3:**
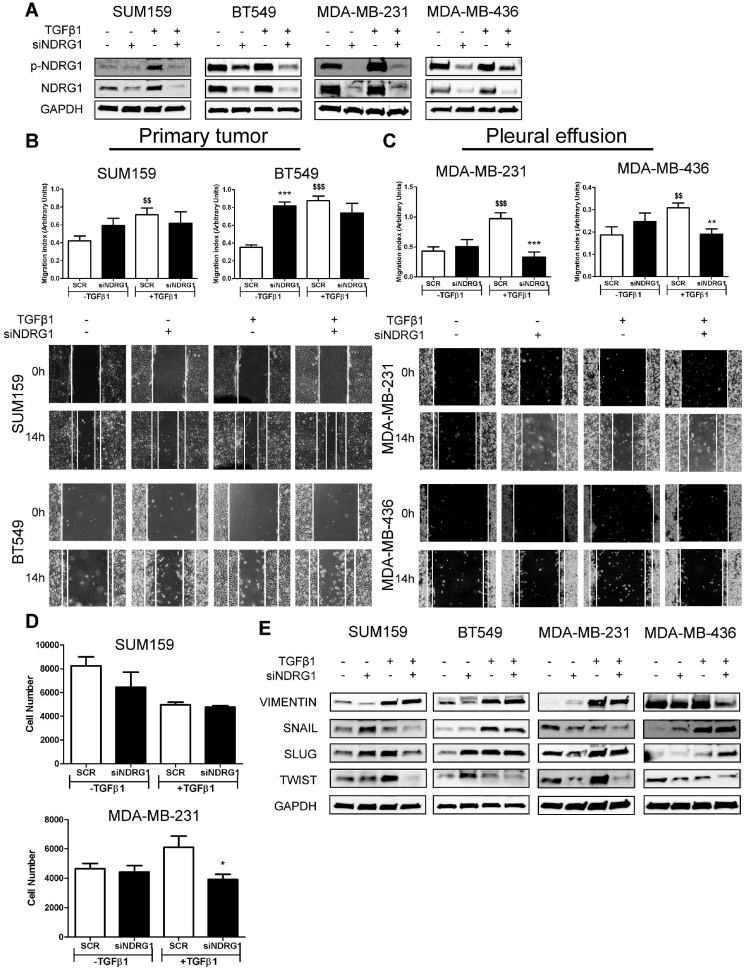
** NDRG1 pleiotropic activity depends on the origin of tumor cells and TGFβ stimulation. A** Western blot of p-NDRG1 (Thr346) and total NDRG1 in SUM159, BT549, MDA-MB-231, and MDA-MB-436 cell lines transfected with siNDRG1 and SCR control with/without TGFβ1 (8+48 protocol). **B** Tumor cell migration of primary-tumor-derived (SUM159, BT549) and **C** pleural-effusion-derived (MDA-MB-231 and MDA-MB-436) cell lines after *NDRG1* knockdown upon stimulation or not with TGFβ1 (8+48 protocol). **D** Tumor cell invasion of SUM159 and MDA-MB-231 cells after *NDRG1* knockdown upon stimulation or not with TGFβ1 (8+48 protocol). **E** Western blot of EMT markers in SUM159, BT549, MDA-MB-231, and MDA-MB-436 cell lines transfected with siNDRG1 or SCR control with/without TGFβ1 (8+48 protocol). * Indicates differences between siNDRG1 and SCR. $ Indicates differences between SCR with and without TGFβ1. * p<0.05; ** p<0.01; *** p<0.001; $$ p<0.01; $$$ p<0.001.

The dual role of NDRG1 as a metastasis/tumor progressor or suppressor has been reported in breast cancer 9,14-18. Inconsistent and variable results in TNBC cell lines have been reported when assessing the role of NDRG1 on proliferation, metastatic abilities, and tumor-initiating cells in TNBC cell lines 9,17,18. To clarify and answer inconsistencies among different reports, and the NDRG1 controversy, and based on our results showing that NDRG1 could be involved in TGFβ signaling pathway, we hypothesized that NDRG1 could exhibit different roles depending on the origin of the TNBC cell lines (established from primary tumor or pleural effusion/metastatic lesion) under stimulation with TGFβ1. To validate this hypothesis, we first investigated the role of NDRG1 on metastatic features of tumor cells. We treated SUM159, BT549 (both established from primary tumor), MDA-MB-231, and MDA-MB-436 (both derived from pleural effusion) with TGFβ1 and, 8h later, the *NDRG1* gene was knocked down for 48h (8+48 protocol). Transfection efficiency was validated in every cell line, as well as the increased expression of NDRG1 and p-NDRG1 (Fig. 3A). As reported before 18, NDRG1 inhibition did not cause a decrease in migration in the four cell lines tested, but it was even enhanced in BT549. When NDRG1 was silenced upon stimulation with TGFβ1, although we did not find reduced migration in cells established from primary tumors (Fig. 3B), a significant decrease was seen in those cells derived from pleural effusion compared to the TGFβ1-treated control group (Fig. 3C). These results were confirmed in Hs578T and MDA-MB-468, derived from primary tumor and pleural effusion, respectively (Fig. S3A). Similarly, tumor cell invasion in Matrigel-coated Boyden chambers was reduced by *NDRG1* knockdown under TGFβ1 treatment only in MDA-MB-231, whereas no changes were detected in SUM159 cells (Fig. 3D). Furthermore, TGFβ signaling is a known EMT driver which is involved in tumor metastasis and the generation of tumor-initiating cells with both stemness properties and chemoresistance 40. Hence, we studied whether NDRG1 could have a differential role on EMT upon TGFβ1 stimulation by western blot in primary-tumor- or pleural-effusion-derived cell lines. As expected for a metastasis suppressor, *NDRG1* knockdown without TGFβ1 had little, or no effect, or enhancing effects on the expression of EMT markers. Nevertheless, with TGFβ1, our results showed that *NDRG1* knockdown correlated with reduced Twist protein levels in all cell lines, while Snail and Slug were also downregulated in SUM159 (Fig. 3E). These results and the absence of effect on migration and invasion in primary-tumor-derived cells suggest that Snail, Slug, or Twist are not affected by NDRG1 as part of a cell program to induce metastasis. In contrast to previous investigations in colorectal and prostate cancer cells 19, Vimentin was inhibited after *NDRG1* silencing in MDA-MB-231 and MDA-MB-436 cells treated with TGFβ1, which was not seen in SUM159 and BT549 (Fig. 3E). These results support the NDRG1 pleiotropy on the same molecules in different cancers as previously hypothesized 8.

**Figure 4:**
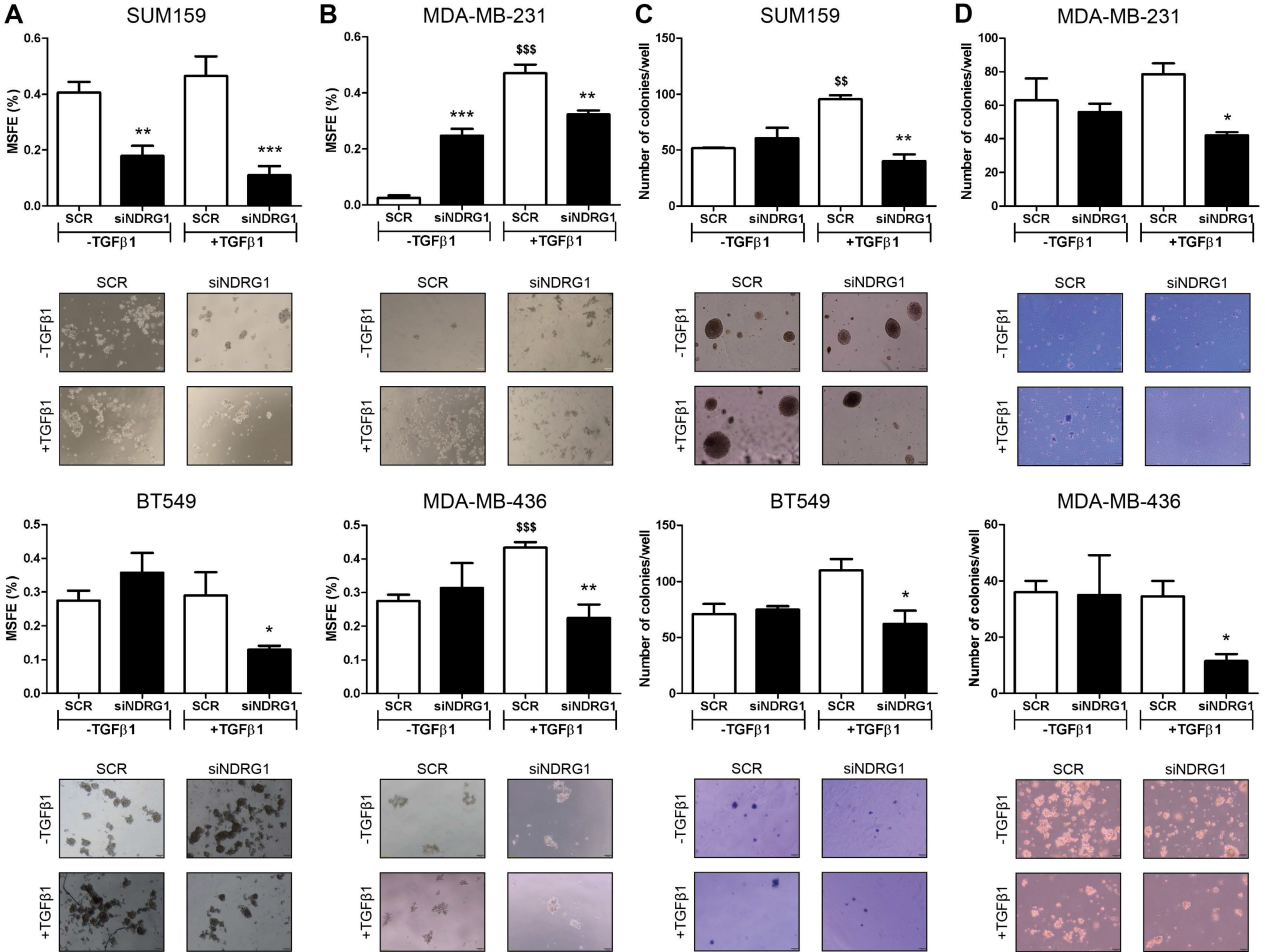
** NDRG1 is involved in the maintenance of TGFβ-induced CSCs. A** Mammosphere-forming efficiency (MSFE) in secondary mammospheres of SUM159 and BT549 cell lines after *NDRG1* inhibition (48h), with/without TGFβ1 for 14 days. **B** MSFE in secondary mammospheres of MDA-MB-231 and MDA-MB-436 cell lines after *NDRG1* inhibition, with/without TGFβ (8+48 protocol). **C** Soft-agar colony formation in SUM159 and BT549 cell lines after *NDRG1* inhibition and treatment with TGFβ (14-day protocol) and **D** in MDA-MB-231 and MDA-MB-436 cell lines after *NDRG1* inhibition and treatment with TGFβ1 (8+48 protocol). * Indicates differences between siNDRG1 and SCR. $ Indicates differences between SCR with and without TGFβ1. * p<0.05; ** p<0.01; *** p<0.001; $$ p<0.01; $$$ p<0.001.

Notably, Twist, which was consistently inhibited by *NDRG1* knockdown in presence of TGFβ1, is not only involved in EMT and metastasis but leads to the generation of cancer stem cells (CSCs), chemoresistance, and tumor progression 41. It is known that a long treatment with TGFβ1 induces CSCs and drug resistance, while a shorter stimulation facilitates lung colonization by breast cancer cells 42. Hence, because NDRG1 was reported to enhance resistance to chemotherapy 43, we hypothesized that NDRG1 could have a role on CSCs by TGFβ1 in TNBC cells derived from primary tumor after long-term exposure to TGFβ1, whereas shorter stimulation would be enough for those cells which are derived from pleural effusion. We tested our hypothesis by treating SUM159 and BT549 cells with TGFβ1 for 14 days, and *NDRG1* knockdown at day 12 (14-day protocol), while MDA-MB-231 and MDA-MB-436 cells were maintained at 8+48 protocol. First, we confirmed that a shorter treatment with TGFβ1 (8+48 protocol) in SUM159 cells did not reveal any effect of *NDRG1* knockdown in the formation of 2MS (Fig. S3B). On the contrary, we found that *NDRG1* inhibition in presence of TGFβ1 caused a significant decrease of 2MS in SUM159, BT549 (Fig. 4A), MDA-MB-231, and MDA-MB-436 cell lines compared to SCR control with TGFβ1 (Fig. 4B). Except for SUM159, 2MS-forming ability was enhanced (BT549) or not changed by *NDRG1* inhibition without TGFβ1.

Similar results were found in the formation of 1MS (Fig. S3C). In CSC-enriched 2MS cultures of SUM159 cells, *NDRG1* knockdown without TGFβ1 triggered the formation of 3MS. As expected, a reduction of MSFE was observed in the 3MS from TGFβ1-stimulated 2MS after *NDRG1* inhibition, both in SUM159 and MDA-MB-231 cells (Fig. S3D). In the same way, soft-agar colony formation provided similar findings as only knockdown of *NDRG1* with TGFβ1 led to a reduced number of colonies in primary-tumor- and pleural-effusion-derived cell lines (Fig. 4C and D). Overall, these results suggest that NDRG1 is mainly involved in the maintenance of TGFβ-induced CSCs, regardless of the origin of cancer cells, and it only has a role in the metastatic ability of cells from metastatic lesions.

**Figure 5:**
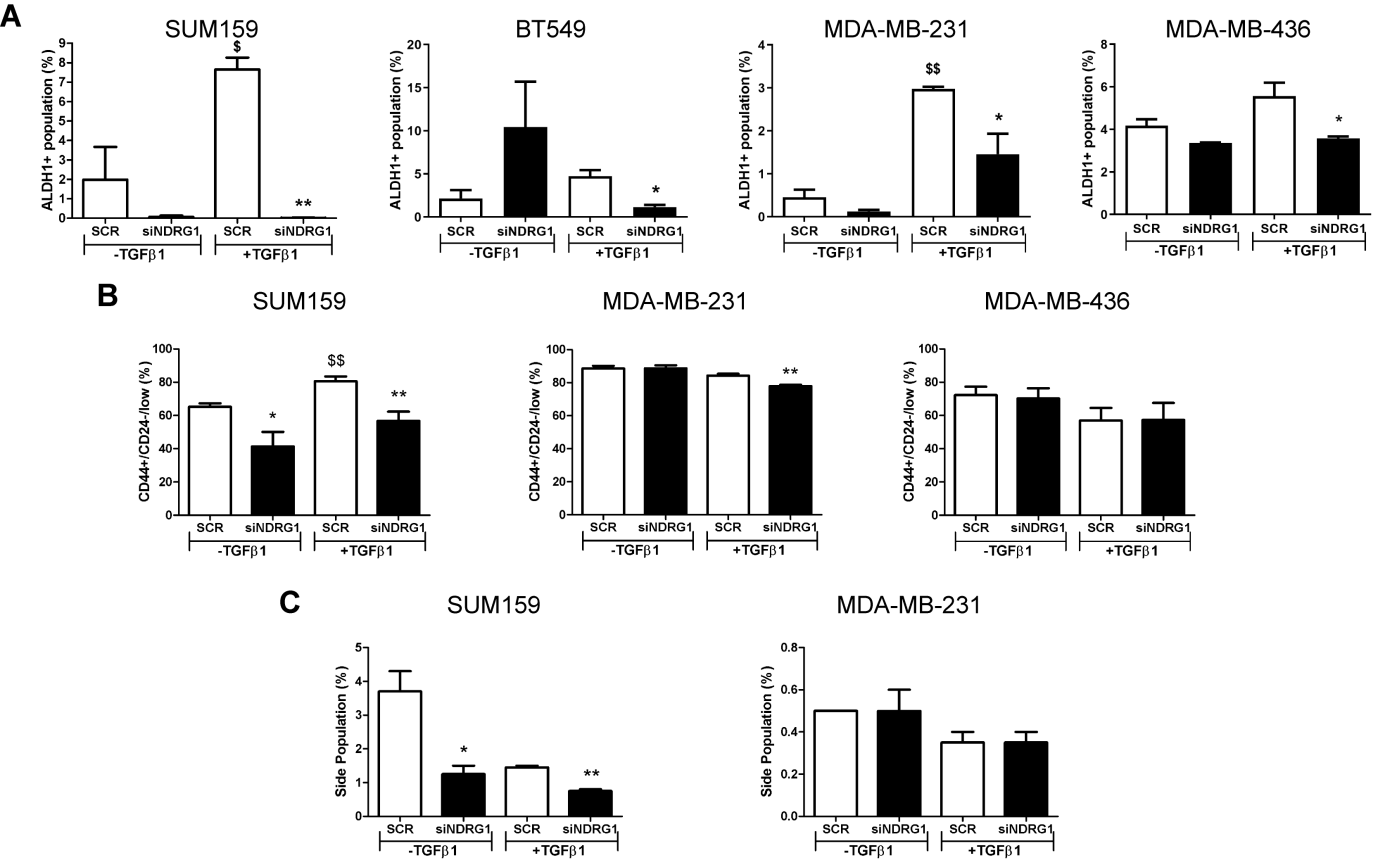
** NDRG1 driven by TGFβ1 has a distinctive role in different types of CSC populations. A** Flow cytometric analysis of ALDH1^+^ CSCs after *NDRG1* knockdown in the presence/absence of TGFβ1 in SUM159, BT549, MDA-MB-231, and MDA-MB-436 cell lines. **B** Flow cytometric analysis of CD44^+^/CD24^low/-^ population in SUM159, MDA-MB-231, and MDA-MB-436 cell lines after *NDRG1* knockdown in the presence/absence of TGFβ1. **C** Side population analysis in SUM159 and MDA-MB-231 cell lines after *NDRG1* knockdown, in the presence/absence of TGFβ1. * Indicates differences between siNDRG1 and SCR. $ Indicates differences between SCR with and without TGFβ1. * p<0.05; ** p<0.01; $ p*<*0.05; $$ p*<*0.01.

Because NDRG1 pleiotropism is not only dependent on the cancer type but also the origin of tumor cells, we next evaluated whether NDRG1 driven by TGFβ1 could have a distinctive role in different types of CSCs populations, namely the chemoresistant, metastatic, and proliferative ALDH1^+^, the more quiescent, chemoresistant, and invasive CD44^+^/CD24^-^, and the drug-resistant side population 44-46. First, we confirmed that the 8+48h protocol did not have any effect on the ALDH1^+^ population in cells from primary tumors (Fig. S4A). Our findings revealed a reduced number of ALDH1^+^ population in all cell lines after *NDRG1* knockdown with TGFβ1, regardless of the origin of the cell line, compared with their corresponding negative controls (Fig. 5A; Fig. S4B). In contrast, an evident diminution of CD44^+^/CD24^-/low^ population was found in SUM159 cells after *NDRG1* knockdown, whereas very little or no change was seen in MDA-MB-231 and MDA-MB-436 cells, respectively (Fig. 5B; Fig. S4C). Similarly, the side population was modified only in SUM159 cells (Fig. 5C; Fig. S4D), although Multi-Drug Resistance 1 (MDR1), or p-glycoprotein, was reduced in both SUM159 and MDA-MB-231 cells only upon activation with TGFβ1 (Fig. S5A), what endorses the notion that NDRG1 is involved in tumor chemoresistance.

Altogether, these data will help to understand the extent of the pleiotropism exhibited by NDRG1 and suggest that it can be involved in the maintenance of different CSCs subpopulations in TNBC, depending on the tissue of origin of the cell lines (primary tumor or pleural effusion), and their grade of differentiation into a more aggressive phenotype induced by TGFβ1.

### Inhibition of TGFβ and GSK3β represents a novel therapeutic approach to target TGFβ1-induced NDRG1 in TNBC

**Figure 6:**
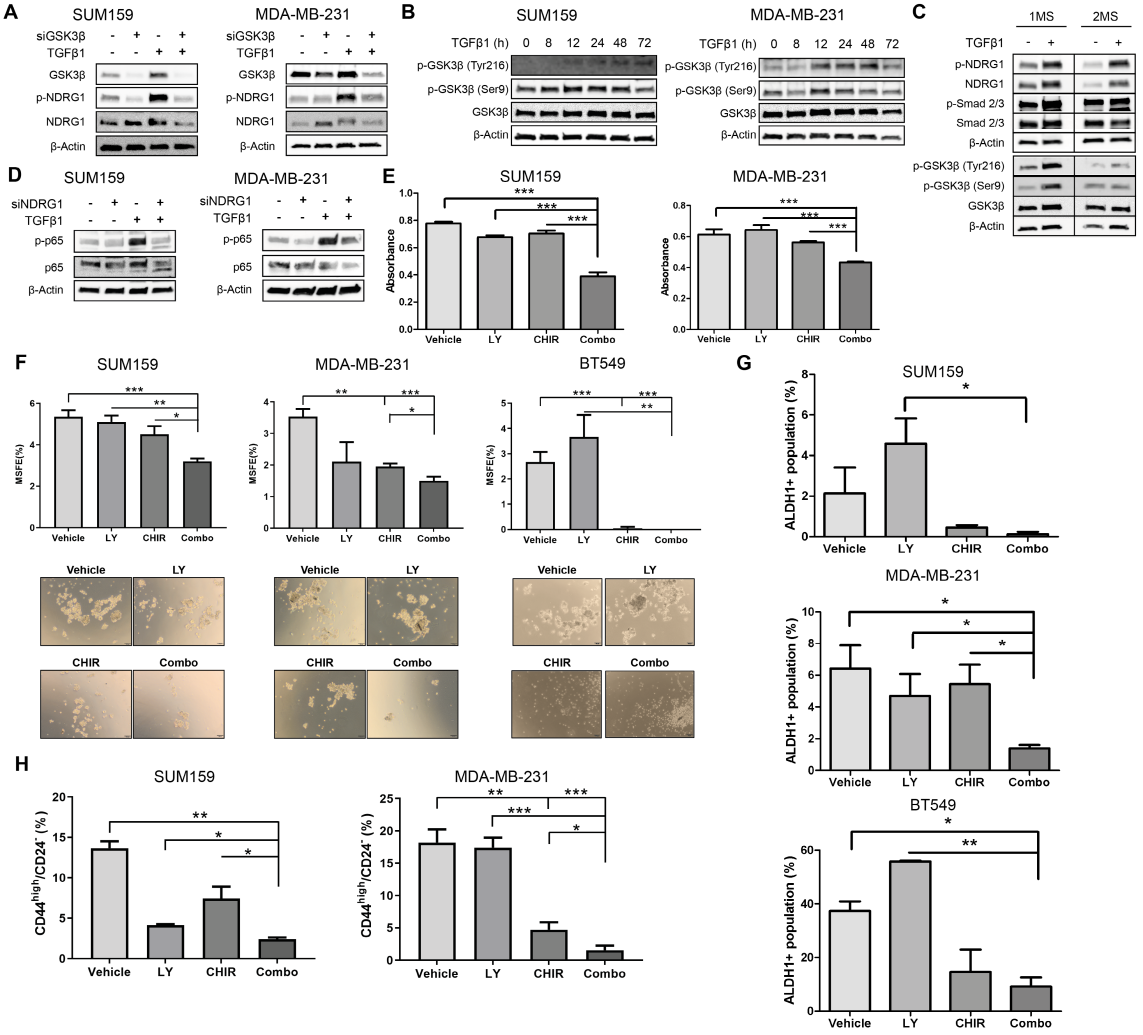
** Pathway-guided identification of a potential treatment of TGFβ1-induced NDRG1 in TNBC. A** Western blot of NDRG1 and p-NDRG1 (Thr346) in cells transfected with siGSK3β, with/without TGFβ1 in SUM159 and MDA-MB-231 cell lines. **B** Protein expression of p-GSK3β (Ser9) and p-GSK3β (Tyr216) after treatment with TGFβ1 at indicated time points in SUM159 and MDA-MB-231 cells. **C** Western blot of NDRG1, p-NDRG1, SMAD2/3, p-SMAD2/3, GSK3β, p-GSK3β in primary (1MS) and secondary mammosphere (2MS) cultures of SUM159 cells, with/without TGFβ1. **D** Western blot of p65 and p-p65 (RelA) in SUM159 and MDA-MB-231 cells after *NDRG1* knockdown in the presence/absence of TGFβ1. **E** Proliferation assay in SUM159 and MDA-MB-231 cell lines in adherence conditions after treatment with TGFβ (LY2157299, LY), GSK3β (CHIR99021, CHIR) inhibitors and their combination (Combo). **F** MSFE in SUM159, MDA-MB-231, and BT549 cell lines in 2MS after treatment with LY, CHIR, and their combination (Combo), previous stimulation with TGFβ1. **G** ALDH1^+^ activity in 2MS of SUM159, MDA-MB-231, and BT549 cell lines after treatment with LY, CHIR, and their combination (Combo), previous stimulation with TGFβ1. **H** CD44^high^/CD24^-^ flow cytometry of 2MS in SUM159 and MDA-MB-231 cell lines after treatment with LY, CHIR, and their combination (Combo), previous stimulation with TGFβ1. * p<0.05; ** p<0.01; *** p*<*0.001.

Being confirmed that NDRG1, p-NDRG1 (Thr346) expression, and subcellular localization is associated with poorer survival of TNBC patients and that it is involved in tumor progression induced by TGFβ signaling, our final goal was to propose a potential way for their personalized treatment. For this reason, we next investigated a plausible signaling pathway upstream and downstream of NDRG1 to rationally identify and design a candidate for targeted therapy. First, based on our results and previous works, we identified potential signaling pathways that modulate NDRG1 activity by pharmacological inhibition of PI3K, Akt, mTOR, SGK1/2, GSK3β, and TGFβ in SUM159 and MDA-MB-231 cells stimulated with TGFβ1 for 24, 48, and 72h. Only those inhibitors that caused a reduction of p-NDRG1 within the same time point (each inhibitor in both cell lines), in at least two time points in both cell lines, were selected for further experiments. According to these criteria, TGFβRI (LY2157299) (at 48 and 72h), mTOR (rapamycin) (at 24 and 72h), and GSK3β (CHIR99021) (at 24 and 48h) (Fig. S5B) were used for further analyses. These results suggest that GSK3β could be a kinase immediately upstream of NDRG1 as previously reported 47. To test that possibility, we knocked down the *GSK3B* gene with siRNA in SUM159 and MDA-MB-231 cells with and without TGFβ1. As expected, our results in absence of TGFβ stimulation demonstrated that GSK3β inhibition enhanced total NDRG1 expression and reduced phosphorylation in both cell lines (Fig. 6A). These findings agree with previous reports showing that the GSK3β kinase activity phosphorylates p-NDRG1 (Thr346) at different sites for instability and proteasomal degradation of NDRG1 as a tumor suppressor 8,32,48. Strikingly, upon stimulation with TGFβ1, we detected accumulated protein levels of GSK3β and its knockdown reduced p-NDRG1 and total NDRG1 in both cell lines (Fig. 6A), which suggests that TGFβ1 can promote a different role of GSK3β on NDRG1 that prevents its degradation. The GSK3β inhibitor CHIR-99021 increases the inhibitory phosphorylation of GSK3β at Ser9 49; however, we observed lower p-NDRG1 levels after treatment with the GSK3β inhibitor. Together, these results suggest that active TGFβ signaling evokes tumorigenic NDRG1 activity through the activation of GSK3β (phosphorylated at Tyr216). We tested our hypothesis and found that treatment with TGFβ1 induced a time-dependent shift from inactive p-GSK3β (Ser9) to active p-GSK3β (Tyr216) that included a coexistence of both forms in a certain moment (Fig. 6B). Because TGFβ-mediated NDRG1 expression was highly correlated with CSCs in our experiments, we validated these results in 1MS and 2MS cultures of SUM159 cells with/without TGFβ1. We found that TGFβ1 upregulated total, p-NDRG1, and p-SMAD2/3 in 1MS and 2MS. Again, TGFβ1 enhanced the levels of active and inactive p-GSK3β that coexisted in 1MS, but only the p-GSK3β active form was higher in 2MS (Fig. 6C). Given that mammosphere subcultures are enriched in CSCs, our results support previous reports showing that active p-GSK3β has an important role in CSCs 50.

Thereafter, we tested the three compounds selected above upon stimulation with TGFβ1 for 24h in SUM159 and MDA-MB-231 cells. Our results confirmed that only TGFβRI (LY2157299, LY) and GSK3β inhibitors (CHIR99021, CHIR) reduced p-NDRG1, and this decrease was enhanced when both molecules were combined (Combo). However, we did not observe a decrease in total NDRG1 expression (Fig. S5C). It was recently reported in cancer cells the existence of two NDRG1 isoforms, at ~47kDa and ~46kDa, which correlated with phosphorylation at Thr346 and Ser330, respectively. NDRG1 processing in cancer cells includes proteasome-dependent processing of the ~47kDa isoform into the ~46kDa isoform that is further degraded by the lysosomal compartment. Interestingly, GSK3β inhibitors were able to reduce the expression of the ~47kDa isoform through lysosomal degradation independently of their kinase inhibitor activity 47, which is in agreement with our observations (Fig. S5C, upper and lower right panels). Because NF-κB is involved in EMT, regulation of Twist, and CSC phenotype, it is interrelated with TGFβ in EMT and CSC dynamics 51,52, and it was found to correlate with NDRG1 as a tumor suppressor and progression promoter in colon and esophageal cancer, respectively 7,53, we hypothesized that it could be a downstream effector of TGFβ-induced NDRG1 in TNBC. We found that p-p65 (RelA) levels were enhanced by TGFβ1 and reduced by *NDRG1* knockdown only in TGFβ1-treated cells (Fig. 6D), which supports our hypothesis and explains the role of NDRG1 on TNBC progression. Similar findings were obtained after single and combined treatment with both TGFβ and GSK3β inhibitors in SUM159 and MDA-MB-231, treated with TGFβ1 for 24 and 72h, respectively (Fig. S5D).

We finally investigated the potential of TGFβ and GSK3β inhibitors as single and combination therapy for the personalized treatment of TNBC patients. First, we found that Combo efficiently reduced tumor cell proliferation in adherence conditions (Fig. 6E). Next, the lowest MSFE was seen with Combo in both 1MS and 2MS in the three cell lines tested (Fig. 6F; Fig. S5E). As described above, our results have shown that NDRG1 is highly involved in CSCs, hence, 2MS cultures (enriched in CSCs) were treated both with the inhibitors and TGFβ1. We observed that the Combo group exhibited the highest activity to reduce ALDH1^+^, although it was only significant compared with single agents in MDA-MB-231 cells (Fig. 6G; Fig. S6A). On the contrary, diminution of CD44^high^/CD24^-^ subpopulation by Combo was significantly higher compared with that shown by single drugs in both cell lines (Fig. 6H; Fig. S6B). Herein, we demonstrated that a combination of TGFβ and GSK3β inhibitors can represent an attractive alternative for the treatment of TNBC patients whose overexpression of NDRG1 is associated with poorer survival.

## Discussion

In this study, we explored the association and influence of the differential expression and subcellular localization of NDRG1 and p-NDRG1 (Thr346) on the survival of patients with TNBC, investigated the role and mechanism of TGFβ as a major inductor of NDRG1 pleiotropy, and proposed a possible therapeutic option. NDRG1 pleiotropy is a known event in several human cancers, although the driving causes are still unknown 3-9. Breast cancer exemplifies this dichotomy, in which NDRG1 suppresses metastasis in ER-positive subtypes and promotes tumor progression in aggressive forms of breast cancer, such as TNBC 9,14-18,26. Moreover, post-translational phosphorylation at Thr346 or Ser330, subcellular localization, and interplay of NDRG1 with other molecules have been postulated as responsible for such a pleiotropy 8,10-13; however, their role in patient survival was not previously assessed. Previous studies reported that high NDRG1 expression correlates with poor patient survival in aggressive, inflammatory, and TNBC by using publicly available gene datasets and/or tumor tissue specimens 9,17,18,26. The investigations carried out by Schlee Villodre 9 on TMAs from 216 breast cancer patients (97 cases of TNBC) after neoadjuvant therapy showed different H-score median as cutoff value (160) to discriminate between high and low NDRG1 expression. This threshold value was 120 in a cohort of 64 inflammatory breast cancer (16 were TNBC) tissue specimens after neoadjuvant treatment by the same research group 26. As seen, different cutoff values were assigned to distinguish between high and low NDRG1 expression in TNBC samples by the same group, which could be even more different among different hospitals and countries. Moreover, whereas previous studies assessed either total NDRG1 expression or subcellular localization to determine patient survival 8,9,26, our study demonstrates that p-NDRG1 (Thr346) is a relevant player that must also be evaluated. These drawbacks of previous investigations are solved herein by just establishing positive or negative staining and subcellular localization of both total NDRG1 and p-NDRG1 (Thr346). We have demonstrated that different combinations correlate with patient survival, and we were able to propose them, in an easier, more standardized, precise, and objective way, as risk factors associated with higher patient mortality in our study cohort (Fig. 2E, F and G). However, a limitation of our study is the size of the patient cohort and the low number of cases after neoadjuvant therapy; therefore, further studies must be done on a larger and independent set of patients to validate whether the proposed patient stratification and risk factors could also be predictive of patient prognosis. Overall, our study includes the second larger cohort of TNBC tumor specimens in which NDRG1 is studied and demonstrates, for the first time, not only that both total NDRG1 and p-NDRG1 must be determined in tumor tissue, but also that their subcellular location and their combinations are important biomarkers to be assessed in TNBC patient tumor specimens by immunohistochemistry, as well as in other breast cancer subtypes or tumor types.

Even within the same TNBC subtype, experimental inconsistencies among different studies suggest that NDRG1 pleiotropy also occurs between different cell types or populations, which could invariably affect patient prognosis and their response to treatments. For example, NDRG1 knockdown reduced the proliferation of MDA-MB-231 and MDA-MB-468, but not of SUM159 17,18, SUM149, or BCX010 9. Also, no effects were observed on the migration of MDA-MB-231 and SUM159 cells 18, and metastatic properties and tumor-initiating cells were inhibited in SUM149 or BCX010 9. The authors of this latter study chose those two cell lines because they are aggressive (namely migratory and are enriched in tumor-initiating cells), however, these reasons do not explain why NDRG1 knockdown did not reduce migration in MDA-MB-231 or SUM159 cells (both being aggressive, metastatic and enriched in tumor-initiating cells) in a different study 18. We found in our patient cohort the correlation between TGFβ1, NDRG1, and p-NDRG1 (Ther346) and, also, we previously observed higher levels of p-NDRG1 (Thr346) induced by TGFβ1 20; therefore, we explored whether NDRG1 could have a distinct cell-type-dependent role on TNBC progression when is modulated by the pleiotropic TGFβ signaling pathway 20,21. In the context of active TGFβ signaling, we found that NDRG1 is only involved in the metastatic abilities of TNBC cells that have previously metastasized to distant tissues, which is supported by the notion that breast tumor cells from pleural effusion have a different gene profile compared to their counterparts in primary tumors and express metastasis-related pathways such as TGFβ 54. Additionally, this event could be due to the reduced expression of vimentin, evoked by siNDRG1 with TGFβ1 only in these types of cell lines, which is associated with tumor cell invasion and metastasis 55. Notably, the inhibition of Twist in all cell lines, as well as Snail and Slug in SUM159, indicates that NDRG1 may not induce metastasis through modulation of TGFβ-induced EMT 40.

Given that TGFβ, Twist, and Vimentin also contribute to the generation of CSCs, drug resistance, and tumor progression 41,42, and because NDRG1 promotes chemoresistance 43, we explored whether TGFβ-driven NDRG1 could have a role on TNBC progression by modulation of CSCs. We found that NDRG1 maintains TGFβ-induced CSCs, specifically ALDH1^+^ in all cell lines and CD44^+^/CD24^-^ and side population in primary-tumor-derived cells. Growing evidence shows that, due to CSC heterogeneity, EMT activation and generation and plasticity of CSCs are tightly interrelated 56. In fact, CSCs could exist in alternative mesenchymal-like (M, CD44^+^/CD24^-^), epithelial-like (E, ALDH1^+^) states as well as intermediate E/M/CSC phenotypes (i.e., CD44^+^/CD24^-^/ALDH1^+^) 57. Breast tumor cells that portray complete E or M states show less self-renewal capacity or cell plasticity, respectively 58, while those with a hybrid phenotype are essential for tumorigenicity in basal breast cancer cells, have higher plasticity, stemness, mammosphere-forming, and self-renewal capacities, and produce progenies of drug-resistant ALDH1^+^ cells 58,59. Moreover, EMT/CSCs dynamics would explain why most circulating tumor cells (CTCs) co-express epithelial, mesenchymal, and CSCs markers 60. Indeed, a high CD44/CD24 ratio and ALDH1^+^ expression were found invariable in primary tumors, CTCs, and distant metastases, suggesting their stability during the development and metastasis of breast cancer 46. Molecular pathways like TGFβ or NF-κB trigger EMT and CSCs dynamics which results in a more effective program to disseminate, colonize distant organs, adapt to the new site, proliferate, and resist therapies 52. In this sense, it is interesting that NDRG1 was found to promote lung colonization of breast tumor cells in cooperation with KIAA1199 as part of the effects of Coco on tumor cell dormancy 61 and to be upregulated in a subpopulation of slow-cycling breast tumor-initiating cells within CTCs. In fact, when *NDRG1* was knocked down, brain metastases from breast cancer were completely abrogated 62. Strikingly, it was found in pancreatic cancer a subset of slow-cycling stem-like cells that exhibits upregulated components of TGFβ signaling and that partially overlaps with ALDH1^+^, CD44^+^/CD24^-^ and CD133^+^ CSCs 63. Taken together, we suggest that NDRG1 could be involved in the EMT/CSCs dynamics, where a sustained exposure to TGFβ1 on tumor cells in the primary tumor site would induce the initial activation of EMT and further maintenance of different CSC phenotypes to promote distant invasiveness and chemoresistance through the generation and maintenance of mixed populations of tumor-initiating cells, including those with slow cycling features (ALDH1^+^, CD44^+^/CD24^-^ and side population). Also, we suggest that once cancer cells have escaped from their primary site and reached distant organs, short exposure to TGFβ1 would be enough to activate NDRG1 and EMT, facilitate colonization, cell proliferation, and chemoresistance by controlling the homeostasis of ALDH1^+^. Nonetheless, although this latter hypothesis must be confirmed by further studies, we demonstrated that NDRG1 has a role in tumor progression led by the TGFβ signaling pathway to modulate EMT, metastatic and tumor-initiating abilities, and distinct CSC populations at different stages of tumor progression.

**Figure 7:**
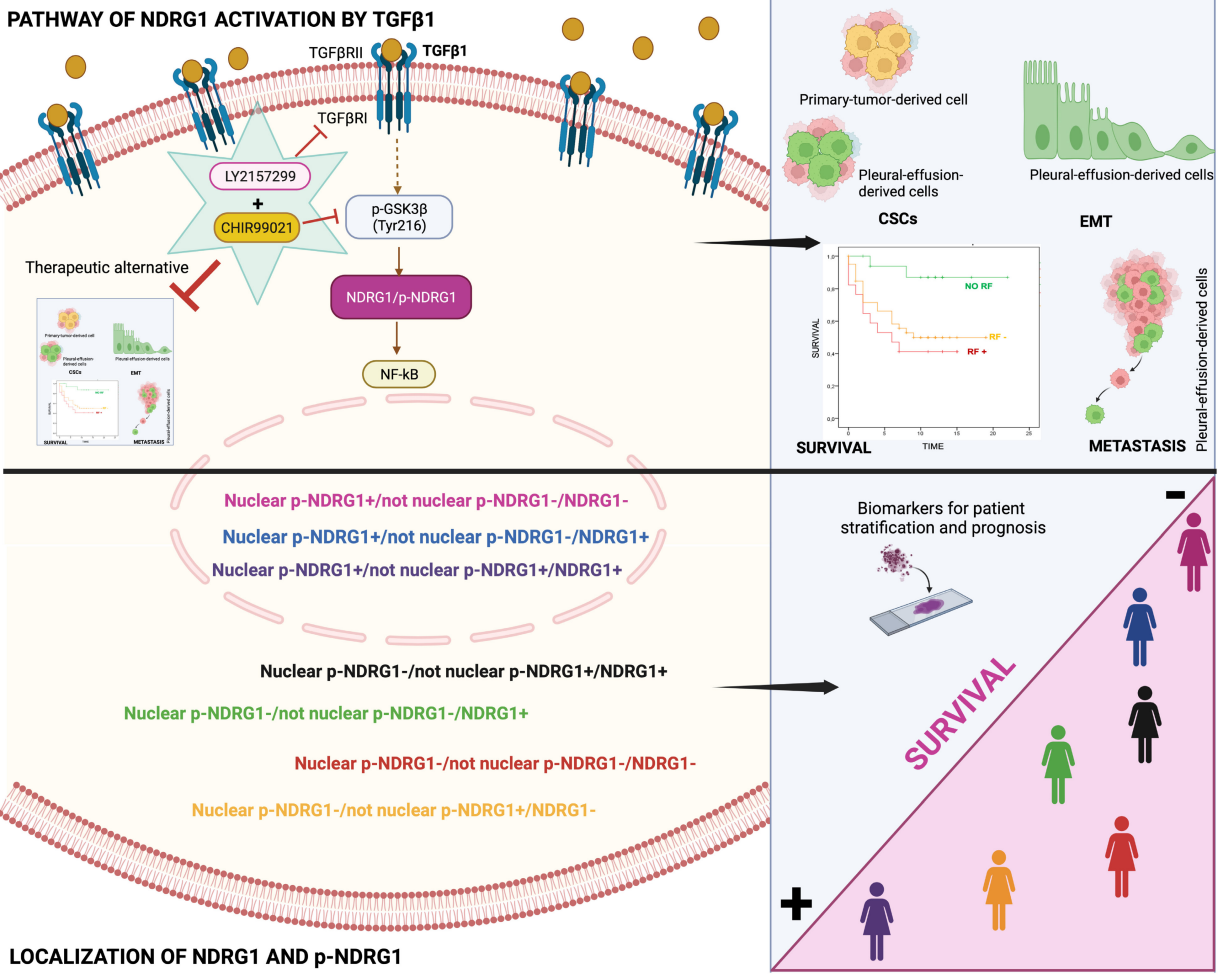
Schematic representation of the signaling pathway of NDRG1 activation mediated by TGFβ1 and GSK3β that is proposed to drive NDRG1 pleiotropy through the modulation of EMT, metastasis, and maintenance of CSCs that renders tumor progression and the survival of TNBC patients. Created with BioRender.com.

Our final goal was to identify the signaling pathway where TGFβ1-induced NDRG1 is involved in to propose a targeted therapy. Our results showed that TGFβRI and GSK3β are located upstream of NDRG1 in the signaling pathway and demonstrated that a combination of TGFβ and GSK3β inhibitors has the potential to reduce TNBC progression by impairing tumor initiation and ALDH1^+^ and CD44^high^/CD24^-^ populations of CSCs. Mechanistically, we found that TGFβ1 induces a shift from inactive to active GSK3β, and it allows the coexistence of both forms in an intermediate time frame (Fig. 7). Similar results were previously reported in MCF10A breast cells, where TGFβ1 induced a shift between active and inactive forms of GSK3α/β, and they coexisted at certain time points. TGFβ1 mainly changed the localization of active forms more than the abundance compared to total GSK3 levels, suggesting that active GSK3α/β accumulates in the endoplasmic reticulum and Golgi apparatus to induce post-translational modifications of newly synthesized proteins 36. In this sense, post-translational modifications of NDRG1, such as phosphorylation or truncation, are events seen in tumor cells that alter the function and localization of NDRG1 8,11. Moreover, in hepatocellular carcinoma, NDRG1 was found to promote tumorigenesis by preventing β-catenin degradation through a direct interaction between NDRG1 and GSK3β 64. However, whether TGFβ-mediated GSK3β activation influences NDRG1 stability by post-translational modifications or direct interaction is unknown. Although, our main goal was not to decipher the mechanism involving NDRG1 stabilization by active GSK3β driven by TGFβ signaling; however, our data provide a different perspective about the positive regulation of NDRG1 by GSK3β to mediate mechanisms of tumor progression driven by TGFβ that must be deeply investigated. Additionally, NF-κB was identified as a possible downstream target of the TGFβ/GSK3β/NDRG1 signaling pathway that could be a final effector on TNBC progression. To our knowledge, there are no previous studies in breast cancer, nor TNBC, that report similar results, but they are supported by previous investigations showing the role of GSK3β in pancreatic tumor progression through activation of NF-κB 65. Overall, our findings suggest that TGFβ can activate GSK3β to stabilize NDRG1, which results in NF-κB activation as an effector of tumor progression. Nevertheless, this signaling pathway must be confirmed by further studies as it was not our goal. Importantly, our results are supported by the hypothesis that NDRG1 has a different interplay with the same molecules in distinct cancers and cell types 8, namely TGFβ1, GSK3β, and NF-κB (Fig. 7).

## Conclusion

In conclusion, our results underline for the first time that both total NDRG1 and p-NDRG1 (Thr346) positiveness status and subcellular location are important biomarkers associated with poor survival of TNBC patients. Those biomarkers could be assessed to stratify these patients, to identify risk factors correlated with poor outcome of the disease, and they could represent biomarkers of patient prognosis. Moreover, this is the first report showing that TGFβ governs the pleiotropic activity of NDRG1 on tumor progression to modulate EMT, metastasis, and tumor-initiating capacity of cancer cells, as well as the maintenance of distinct heterogeneous CSCs populations at different stages of tumor progression. We have also identified that NDRG1 is downstream of TGFβ-induced GSK3β, and our results suggest that the role of NDRG1 in tumor promotion could be attributed to the modulation of NF-κB (Fig. 7). Finally, we propose a therapeutic alternative for TNBC patients, whose overexpression of NDRG1 is associated with poorer survival and TGFβ1 status, by the combination of TGFβ and GSK3β inhibitors to potentially preventing tumor progression, recurrence, and chemoresistance.

## Supplementary Material

Supplementary figures and tables.

## Figures and Tables

**Table 1:** Clinical and pathologic characteristics of the patient cohort

Variable	No. of patients	% of total	Mean survival (years)	p-value (Log-rank)
*Age at diagnosis (years) (median, 58 years) (total n = 83)*				
<58	38	45.8	13.91	0.912
≥58	45	54.2	9.77	
*Age at diagnosis (years) (ROC^1^ ≥73) (total n = 83)*				
<73	67	80.7	14.75	0.037*
≥73	16	19.3	6.92	
*Histological type (total n = 83)*				
Invasive ductal carcinoma (IDC)	73	88	13.71	0.855
Invasive lobular carcinoma (ILC)	2	2.4	5.50	
Other	8	9.6	12.72	
*Tumor grade (total n = 63)*				
I	1	1.6		0.624
II	19	30.2	7.84	
III	43	68.2	12.74	
*Tumor size (total n = 74)*				
T0	2	2.7		0.003*
T1	31	41.9	12.89	
T2	36	48.6	13.65	
T3-4	5	6.8	1.80	
Missing	9			
*Lymph node status (total n = 78)*				
Negative	56	71.8	14.95	0.562
Positive	22	28.2	9.22	
Missing	5			
*Vascular invasion (total n = 54)*				
No	51	94.4	13.38	0.015*
Yes	3	5.6	2.66	
Missing	29			
*Prominent inflammation (total n = 71)*				
No	58	81.7	13.38	0.141
Yes	13	18.3	15.69	
*Ki67 (total n = 82)*				
0-30	36	43.9	14.33	0.896
31-60	31	37.8	12.03	
>60	15	18.3	11.40	
Missing	1			
*Treatment (total n = 79)*				
Neoadjuvant chemotherapy	17	21.5	6,02	0.004*
Adjuvant chemotherapy	51	64.6	16,69	
Other therapy	11	13.9	7,39	
Missing	4			

^1^According to Youden's index.

**Table 2:** Clinical evolution, NDRG1 and p-NDRG1 (Thr346) staining, and univariate analysis

Variable	Number of patients	% of total	Estimated mean survival (years)	p-value (Log-rank)	HR	Cox regression (95% CI)	p-value
*Status (total n = 83)*							
Alive without disease	44	53					
Alive with disease	4	4.8					
Dead by disease	35	42.2					
*Survival time after diagnosis*			13.76				
*NDRG1 (total n = 75)*							
Positive	56	74.7	13.58	0.633	1.00	Ref.	0.643
Negative	19	25.3	13.19		0.81	0.35-1.9	
Missing	8						
*Nuclear*							
Positive	17	22.7	9.41	0.070	1.94	0.91-4.13	0.084
Negative	58	77.3	16.60		1.00	Ref.	
*Cytoplasmic*							
Positive	50	66.7	13.32	0.465	1.32	0.6-2.87	0.479
Negative	25	33.3	13.26		1.00	Ref.	
*Membrane*							
Positive	27	36	11.73	0.437	1.33	0.62-2.83	0.452
Negative	48	64	14.98		1.00	Ref.	
*p-NDRG1 (total n = 77)*							
Positive	56	72.7	13.56	0.981	1.00	Ref.	0.981
Negative	21	27.3	12.14		0.99	0.46-2.13	
Missing	6						
*Nuclear*							
Positive	31	40.3	17.00	0.015*	1.00	Ref.	0.023*
Negative	46	59.7	10.03		2.52	1.13-5.61	
*Cytoplasmic*							
Positive	51	66.2	14.30	0.345	1.00	Ref.	0.362
Negative	26	33.8	10.90		1.38	0.68-2.78	
*Membrane*							
Positive	3	3.9	ND	NA	NA	NA	NA
Negative	74	96.1	13.20				

ND: No Deceased. NA: Not Available. HR: Hazard Ratio.

**Table 3:** Spearman's rank correlation coefficient between NDRG1, p-NDRG1 (Thr346), TGFβ1, and p-GSK3β (Tyr216) staining

		TGFβ1	TGFβ1 (M)	TGFβ1 (C)	TGFβ1 (N)	NDGR1	NDGR1 (M)	NDGR1 (C)	NDGR1 (N)	p-NDGR1	p-NDGR1 (M)	p-NDGR1 (C)	p-NDGR1 (N)	p-GSK3β	p-GSK3β (C)	p-GSK3β (N)
TGFβ1	ρ	*1.000*	***0.247***	***0.992***	*0.083*	*0.205*	*0.060*	*0.182*	***0.282***	*0.128*	*0.050*	*0.145*	*0.020*	*0.092*	*0.094*	*0.053*
	P		0.042*	<0.001*	*0.503*	*0.092*	*0.625*	*0.135*	0.019*	*0.298*	*0.688*	*0.239*	*0.869*	*0.453*	*0.443*	*0.664*
TGFβ1 (M)	ρ		*1.000*	*0.213*	***0.356***	*0.218*	*0.099*	***0.262***	*0.190*	*0.220*	*-0.105*	***0.253***	*0.147*	*-0.009*	*0.020*	*-0.139*
	P			*0.081*	0.003*	*0.073*	*0.420*	0.031*	*0.121*	*0.074*	*0.396*	0.039*	*0.236*	*0.945*	*0.872*	*0.258*
TGFβ1 (C)	ρ			*1.000*	*0.088*	***0.259***	*0.055*	*0.232*	***0.286***	*0.195*	*0.048*	*0.213*	*0.059*	*0.110*	*0.115*	*0.047*
	P				*0.477*	0.033*	*0.654*	*0.057*	0.018*	*0.113*	*0.697*	*0.083*	*0.634*	*0.370*	*0.352*	*0.706*
TGFβ1 (N)	ρ				*1.000*	*-0.018*	*0.167*	*-0.074*	*-0.071*	*-0.079*	*-0.068*	*-0.050*	*-0.023*	*-0.181*	***-0.248***	*0.108*
	P					*0.886*	*0.173*	*0.550*	*0.563*	*0.526*	*0.586*	*0.690*	*0.852*	*0.139*	0.041*	*0.379*
NDGR1	ρ					*1.000*	***0.447***	***0.823***	***0.525***	***0.660***	*0.002*	***0.630***	***0.473***	***0.264***	***0.320***	*0.012*
	P						<0.001*	<0.001*	<0.001*	<0.001*	*0.987*	<0.001*	<0.001*	0.025*	0.006*	*0.918*
NDGR1 (M)	ρ						*1.000*	*0.153*	*0.037*	***0.260***	***0.251***	***0.287***	*0.210*	*0.085*	*0.142*	*-0.014*
	P							*0.190*	*0.756*	0.026*	*0.031*	0.013*	*0.072*	*0.477*	*0.234*	*0.907*
NDGR1 (C)	ρ							*1.000*	***0.344***	***0.628***	*-0.126*	***0.592***	***0.472***	***0.296***	***0.310***	*0.094*
	P								0.002*	<0.001*	*0.284*	<0.001*	<0.001*	0.011*	0.008*	*0.433*
NDGR1 (N)	ρ								*1.000*	***0.424***	*0.041*	***0.285***	***0.410***	*0.150*	*0.159*	*0.045*
	P									<0.001*	*0.727*	0.014*	<0.001*	*0.209*	*0.181*	*0.710*
p-NDGR1	ρ									*1.000*	*0.161*	***0.904***	***0.682***	***0.468***	***0.510***	*0.127*
	P										*0.162*	<0.001*	<0.001*	<0.001*	<0.001*	*0.291*
p-NDGR1 (M)	ρ										*1.000*	*0.109*	*0.094*	*0.178*	*0.163*	*0.150*
	P											*0.346*	*0.418*	*0.137*	*0.175*	*0.213*
p-NDGR1 (C)	ρ											*1.000*	***0.444***	***0.395***	***0.463***	*-0.051*
	P												<0.001*	0.001*	<0.001*	*0.673*
p-NDGR1 (N)	ρ												*1.000*	***0.470***	***0.413***	***0.329***
	P													<0.001*	<0.001*	0.005*
p-GSK3β	ρ													*1.000*	***0.952***	***0.314***
	P														<0.001*	0.007*
p-GSK3β (C)	ρ														*1.000*	*0.122*
	P															*0.307*
p-GSK3β (N)	ρ															*1.000*
	P															

ρ: Spearman's rank correlation coefficient; P: bilateral significance; M: membrane; C: cytoplasm; N: nucleus. Bold numbers indicate statistically significant ρ.

**Table 4:** Paired comparison between groups of patient stratification

		2		3		4		5		6		7	
		Χ^2^	P	Χ^2^	P	Χ^2^	P	Χ^2^	P	Χ^2^	P	Χ^2^	P
Log-Rank (Mantel-Cox)	1	3.069	0.080	0.822	0.365	3.153	0.076	14.606	<0.001*	0.101	0.751	0.233	0.629
	2			1.065	0.302	0.278	0.598	0.104	0.747	2.267	0.132	4.000	0.046*
	3					0.583	0.445	4.784	0.029*	0.707	0.401	0.863	0.353
	4							2.134	0.144	2.440	0.118	1.831	0.176
	5									8.508	0.004*	6.591	0.010*
	6											0.154	0.695

**1:** Nuclear p-NDRG1^-^/not nuclear p-NDRG1^+^/NDRG1^+^ (n=19) **2:** Nuclear p-NDRG1^-^/not nuclear p-NDRG1^+^/NDRG1^-^ (n=4) **3:** Nuclear p-NDRG1^-^/not nuclear p-NDRG1^-^/NDRG1^+^ (n=7) **4:** Nuclear p-NDRG1^-^/not nuclear p-NDRG1^-^/NDRG1^-^ (n=14) **5:** Nuclear p-NDRG1^+^/not nuclear p-NDRG1^+^/NDRG1^+^ (n=25) **6:** Nuclear p-NDRG1^+^/not nuclear p-NDRG1^-^/NDRG1^+^ (n=4) **7:** Nuclear p-NDRG1^+^/not nuclear p-NDRG1^-^/NDRG1^-^ (n=1) Bold numbers indicate statistically significant values.

**Table 5:** Univariate and multivariate Cox regression model of RF^+/-^ on overall survival

Covariate	Univariate				Multivariate			
	N	HR	95% CI	p-value	N	HR	95% CI	p-value
*Risk Factor (RF)*	74			0.044*	62			0.024*
No RF	17	1.00	Ref.		14	1.00	Ref.	
RF^+^	17	6.97	1.52-31.88	0.012*	15	18.19	2.08-158.79	0.009*
RF^-^	40	5.07	1.18-21.78	0.029*	33	8.75	1.09-69.67	0.040*
*Treatment*	79			0.009*	62			0.001*
Neoadjuvant	17	1.00	Ref.		9	1.00	Ref.	
Adjuvant	51	0.29	0.13-0.65	0.003*	42	0.12	0.04-0.38	<0.001*
Other	11	0.69	0.25-1.89	0.479	11	0.15	0.03-0.73	0.020*
*Age (≥73years)*	83	2.09	1.00-4.37	0.050*	62	4.13	1.27-13.43	0.018*
*Tumor size*	74			0.026*				
T0	2	NA	NA	NA				
T1	31	0.18	0.05-062	0.007*				
T2	35	0.16	0.04-0.53	0.003*				
T3-T4	5	1.00	Ref.					
*Prominent inflammation (Yes)*	71	0.42	0.12-1.41	0.163				

N: number of patients; HR: Hazard Ratio; NA: not available (not possible to calculate) RF^+^: [(p-NDRG1(N)^+^ / NDRG1(N)^+^) / (p-NDRG1(N)^-^ / NDRG1(N)^+^)] RF^-^: p-NDRG1(N)^-^ / NDRG1(N)^-^.

**Table 6:** Univariate and multivariate Cox regression analysis of 1RF^+/-^ and 2RF on overall survival

Covariate	Univariate				Multivariate			
	N	HR	95% CI	p-value	N	HR	95% CI	p-value
*Risk Factor (RF)*	74			0.011*	62			0.030*
No RF	17	1.00	Ref.		14	1.00	Ref.	
1RF^+^	13	5.01	1.01-24.87	0.048*	12	14.01	1.48-131.84	0.021*
1RF^-^	40	5.10	1.18-21.95	0.028*	33	8.49	1.07-67.37	0.043*
2RF	4	18.07	3.20-101.90	0.001*	3	30.67	2.93-320.32	0.004*
*Treatment*	79			0.009*	62			0.003*
Neoadjuvant	17	1.00	Ref.		9	1.00	Ref.	
Adjuvant	51	0.29	0.13-0.65	0.003*	42	0.13	0.04-0.44	0.001*
Other	11	0.69	0.25-1.89	0.479	11	0.15	0.03-0.74	0.020*
*Age (*≥*73years)*	83	2.09	1.00-4.37	0.050*	62	4.17	1.27-13.62	0.018*
*Tumor size*	74			0.026*				
T0	2	NA	NA	NA				
T1	31	0.18	0.05-062	0.007*				
T2	35	0.16	0.04-0.53	0.003*				
T3-T4	5	1.00	Ref.					
*Prominent inflammation (Yes)*	71	0.42	0.12-1.41	0.163				

N: number of patients; HR: Hazard Ratio; NA: not available (not possible to calculate). 1RF^+^: p-NDRG1(N)^+^ / NDRG1(N)^+^ RF^-^: p-NDRG1(N)^-^ / NDRG1(N)^-^ 2RF: p-NDRG1(N)^-^ / NDRG1(N)^+^.

## Abbreviations

1MS: Primary mammospheres
2MS: Secondary mammospheres
3MS: Tertiary mammospheres
ALDH1: Aldehyde dehydrogenase 1
CHIR: CHIR99021
CI: Confidence Interval
CSCs: Cancer stem cells
CTCs: Circulating tumor cells
EMT: Epithelial-mesenchymal transition
ER: Estrogen receptor
HR: Hazard Ratio
LN: Lymph node
LY: LY2157299
MSFE: Mammosphere-forming efficiency
NDRG1(C): Cytoplasmic NDRG1
NDRG1(N): Nuclear NDRG1
NDRG1: N-Myc downstream-regulated gene 1
p-NDRG1(C): Cytoplasmic p-NDRG1
p-NDRG1(N): Nuclear p-NDRG1
RF: Risk Factor
RFS: Relapse-free survival
ROC: Receiver operating characteristic
RT: Room temperature
TGFβ: Transforming growth factor β
TMAs: Tissue microarrays
TNBC: Triple-negative breast cancer
