# Supplementary material for: TGFβ Governs the Pleiotropic Activity of NDRG1 in Triple-Negative Breast Cancer Progression
Source: Int J Biol Sci. 2023 Jan 1;19(1):204–24. doi: 10.7150/ijbs.78738 (PMC9760438; doi:10.7150/ijbs.78738)
Supplement: Supplementary file 1 — Supplementary figures and tables. [file ijbsv19p0204s1.pdf]

Supplementary Materials

TGFβ Governs the Pleiotropic Activity of NDRG1 in Triple-Negative Breast Cancer Progression.

Araceli López-Tejada[1,2,3,†], Carmen Griñán-Lisón[2,3,4,†], Adrián González-González[2,†], Francisca E. Cara[2], Rafael J. Luque[5], Carmen Rosa-Garrido[6], José L. Blaya-Cánovas[2,3,4], Alba Navarro-Ocón[2,3], María Valenzuela-Torres[2], Marisa Parra-López[2], Jesús Calahorra[2,3,4], Isabel Blancas[3,7], Juan A. Marchal[3,8,9,#], Sergio Granados-Principal[1,2,3,#,*].

[1]Department of Biochemistry and Molecular Biology 2, School of Pharmacy, University of Granada, 18011 Granada, Spain.

[2]GENYO, Centre for Genomics and Oncological Research, Pfizer/University of Granada/Andalusian Regional Government, 18016 Granada, Spain.

[3]Instituto de Investigación Biosanitaria ibs.GRANADA, University Hospitals of Granada-University of Granada, Spain; Conocimiento s/n 18100, Granada. Spain.

[4]UGC de Oncología Médica, Hospital Universitario de Jaén, 23007 Jaén, Spain.

[5]UGC de Anatomía Patológica, Hospital Universitario de Jaén, Jaén, Spain.

[6]FIBAO, Hospital Universitario de Jaén, Servicio Andaluz de Salud, Jaén, Spain.

[7]UGC de Oncología, Hospital Universitario "San Cecilio", 18016 Granada, Spain

[8]Department of Human Anatomy and Embryology, Biopathology and Regenerative Medicine Institute (IBIMER), University of Granada, 18011 Granada, Spain.

[9]Excellence Research Unit "Modeling Nature" (MNat), University of Granada, Spain.

*Corresponding author. E-mail: sergiogp@ugr.es. Phone number: +34 651 55 79 21

[†] These authors contributed equally to this work.

#Co-senior authors.

Supplementary figures.

Supplementary Figure S1

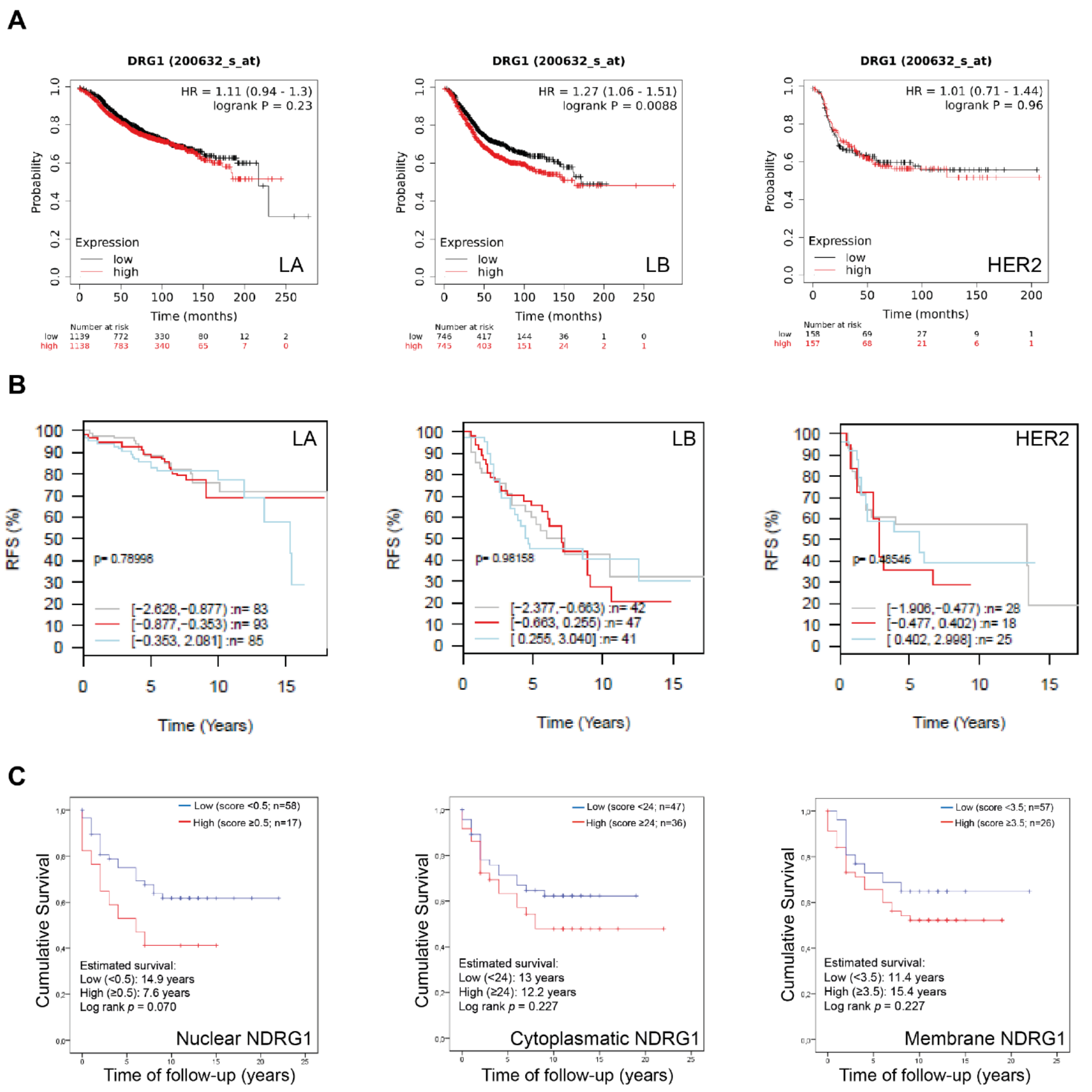

Fig. S1. NDRG1 expression and patient survival in other breast cancer subtypes. A Kaplan-Meier curves with the KM plotter and **B** GOBO databases of high *NDRG1* expression and relapse-free survival (RFS) in luminal A (LA), luminal B (LB), and HER2^{+} breast cancer subtypes. **C** Correlation of NDRG1 expression with cumulative survival of TNBC patients by Kaplan-Meier according to the subcellular localization.

Supplementary Figure S2

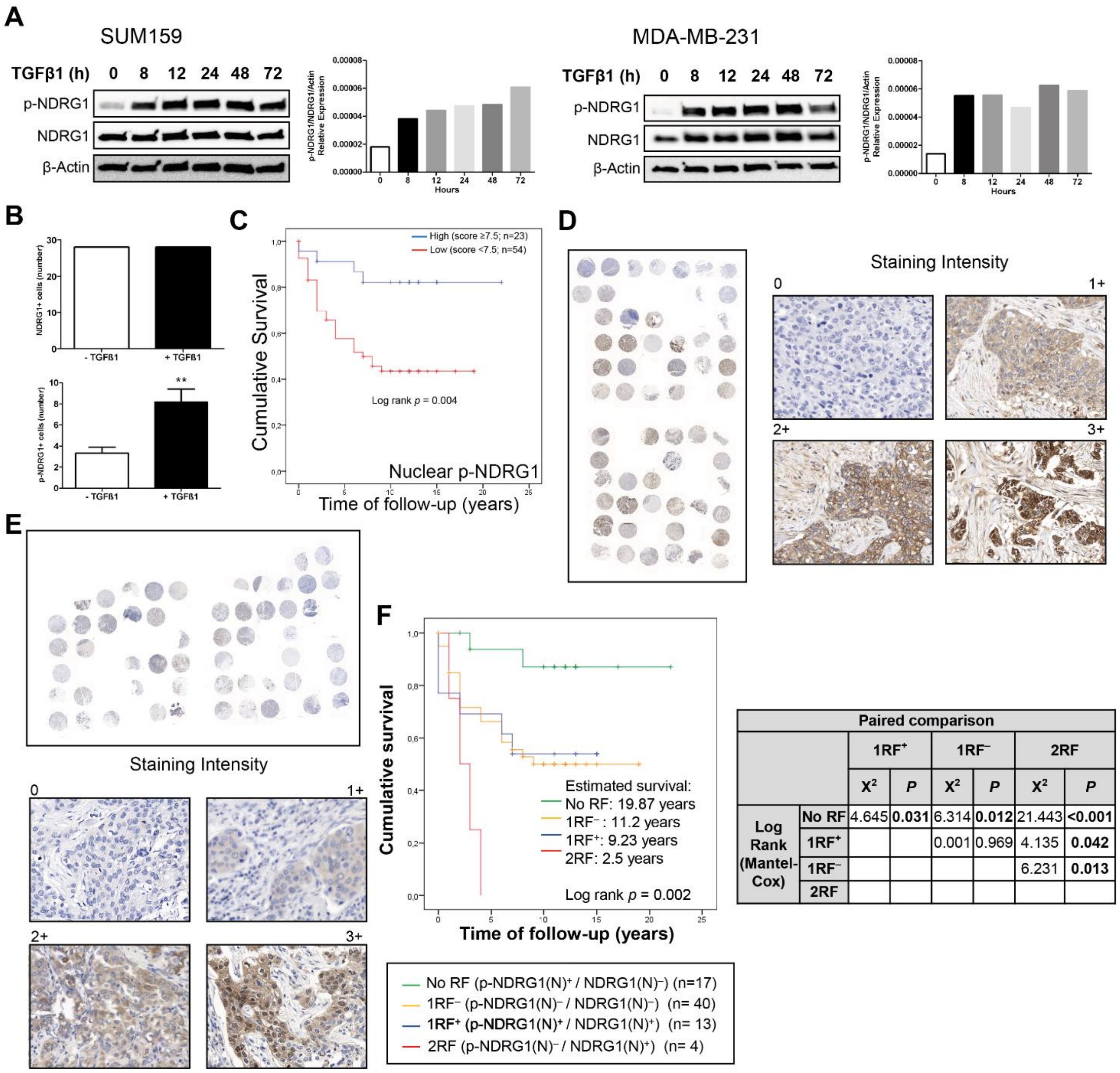

Paired comparison		1RF⁺		1RF⁻		2RF	
		X^2	P	X^2	P	X^2	P
Log Rank (Mantel-Cox)	No RF	4.645	**0.031**	6.314	**0.012**	21.443	**<0.001**
	1RF⁺			0.001	0.969	4.135	**0.042**
	1RF⁻					6.231	**0.013**
	2RF						

Fig. S2. Association of NDRG1 and p-NDRG1 (Thr346) with TGFβ, p-GSK3β (Tyr216), and survival of patients with TNBC. A Western blot analysis of NDRG1 and p-NDRG1 (Thr346) in SUM159 and MDA-MB-231 cell lines after treatment with TGFβ1 at indicated time points. **B** Number of MDA-MB-231 cells that express NDRG1 and p-NDRG1 with or without TGFβ1 stimulation. **C** Impact of nuclear p-NDRG1 (Thr346) staining score (<7.5 and ≥7.5) of TNBC patients' tumor tissue (n=77). **D** Representative images of negative, 1+, 2+, and 3+ TGFβ1 staining intensity in TNBC patients' tumor tissue (original optical objective: 40×). **E** Representative images of negative, 1+, 2+, and 3+ p-GSK3β (Tyr216) staining intensity in TNBC patients'

tumor tissue (original optical objective: 40×). **F** Kaplan-Meier analysis of p-NDRG1(N) and NDRG1(N) staining status as risk factors of shorter cumulative survival after diagnosis of TNBC patients. No Risk Factor (RF) (p-NDRG1(N)$^{+}$/NDRG1(N)$^{-}$), 1RF^{-} (p-NDRG1(N)$^{-}$/NDRG1(N)$^{-}$), 1RF^{+} (p-NDRG1(N)$^{+}$/NDRG1(N)$^{+}$), and 2RF (p-NDRG1(N)$^{-}$/NDRG1(N)$^{+}$), and paired comparison by Chi-square test (n=74). * Indicates differences between -TGFβ1 and +TGFβ1. ** p<0.01.

Supplementary Figure S3

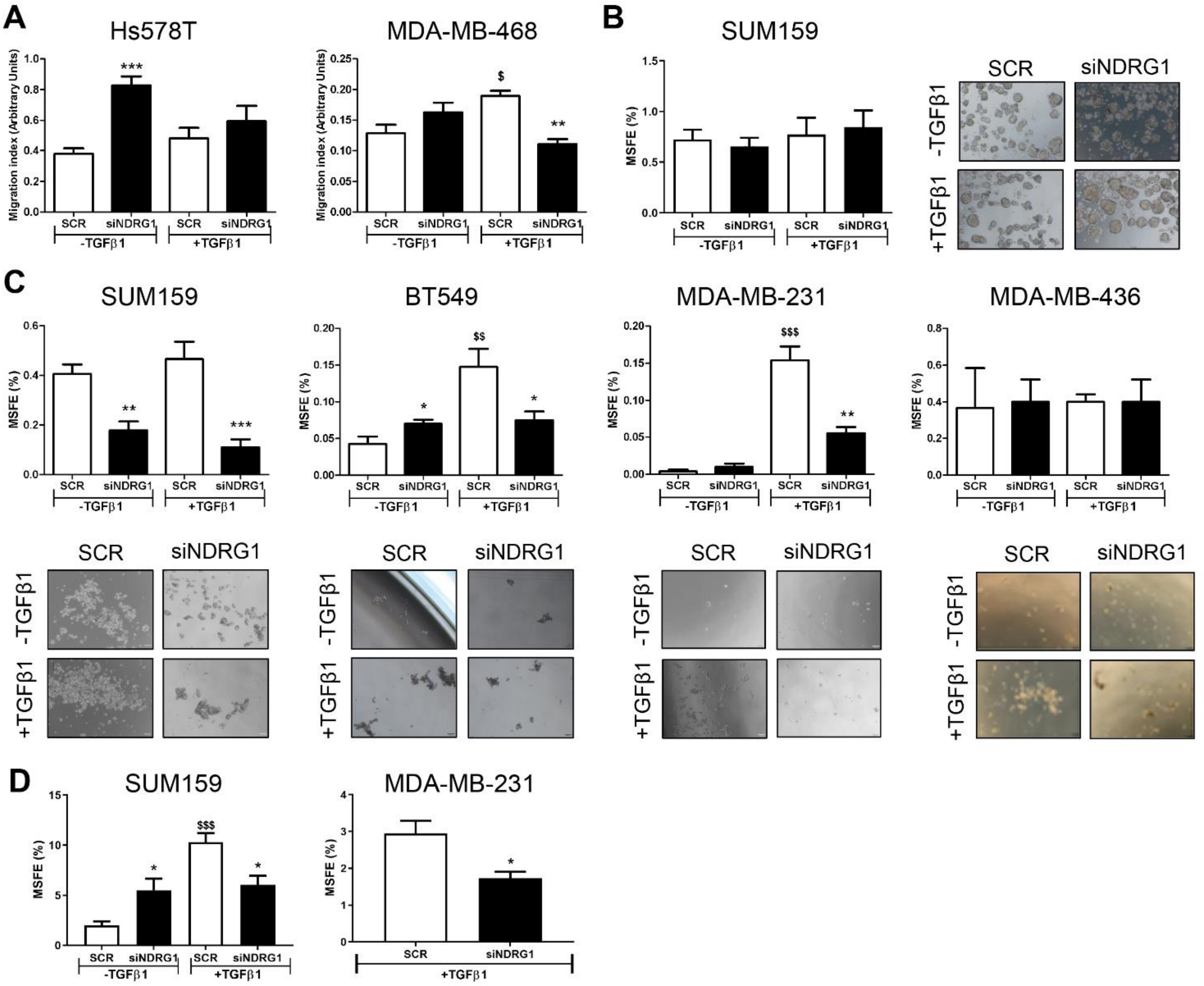

Fig. S3. NDRG1 and TGFβ-induced CSCs. A Migration assay in Hs578T and MDA-MB-468, derived from primary tumor and pleural effusion, respectively, treated with TGFβ1 and siNDRG1 (8+48 protocol). **B** Mammosphere-forming efficiency (MSFE) in secondary mammospheres of SUM159 cells by *NDRG1* knockdown treated with TGFβ1 (8+48 protocol). **C** Primary mammospheres (1MS) in SUM159, BT549 (14-day protocol), MDA-MB-231, and MDA-MB-436 cells (8+48 protocol) after *NDRG1* knockdown,

with/without treatment with TGFβ1. **D** Tertiary mammospheres (3MS) of SUM159 (14-day protocol) and MDA-MB-231 cells (8 +48 protocol) after NDRG1 knockdown, with/without TGFβ1 treatment. * Indicates differences between siNDRG1 and SCR. $ Indicates differences between SCR with and without TGFβ1. * p<0.05; ** p<0.01; *** p<0.01; $ p<0.05; $$ p<0.01; $$$ p<0.001.

Supplementary Figure S4

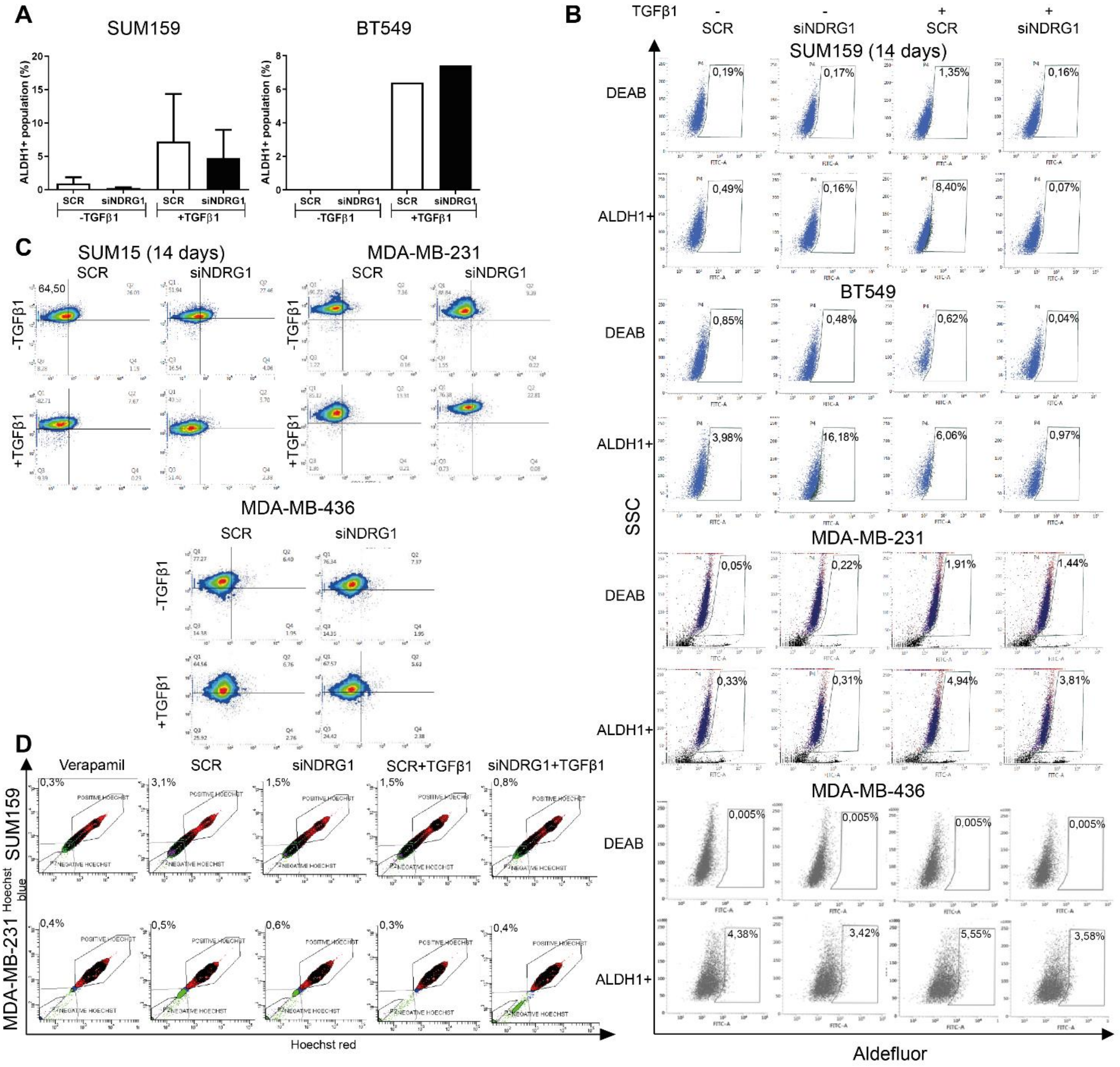

Fig. S4. Short-term protocol of NDRG1+TGFβ1 in primary-tumor-derived cell lines and flow cytometry plots. **A** Flow cytometric analysis of ALDH1$^+$ cell population after *NDRG1* knockdown and TGFβ1 in SUM159 and BT549 cell lines. **B** Dot plots of flow cytometric analysis of aldefluor-positive (ALDH1$^+$)

population and DEAB control in the four cell lines tested after *NDRG1* knockdown and TGFβ1 treatment. **C** Dot plots of flow cytometric analysis of CD44^{high}/CD24^{-} population in SUM159, MDA-MB-231, and MDA-MB-436 cell lines after *NDRG1* knockdown, with/without TGFβ1. **D** Dot plots of side population in SUM159 and MDA-MB-231 cell lines after *NDRG1* knockdown, with/without TGFβ1.

Supplementary Figure S5

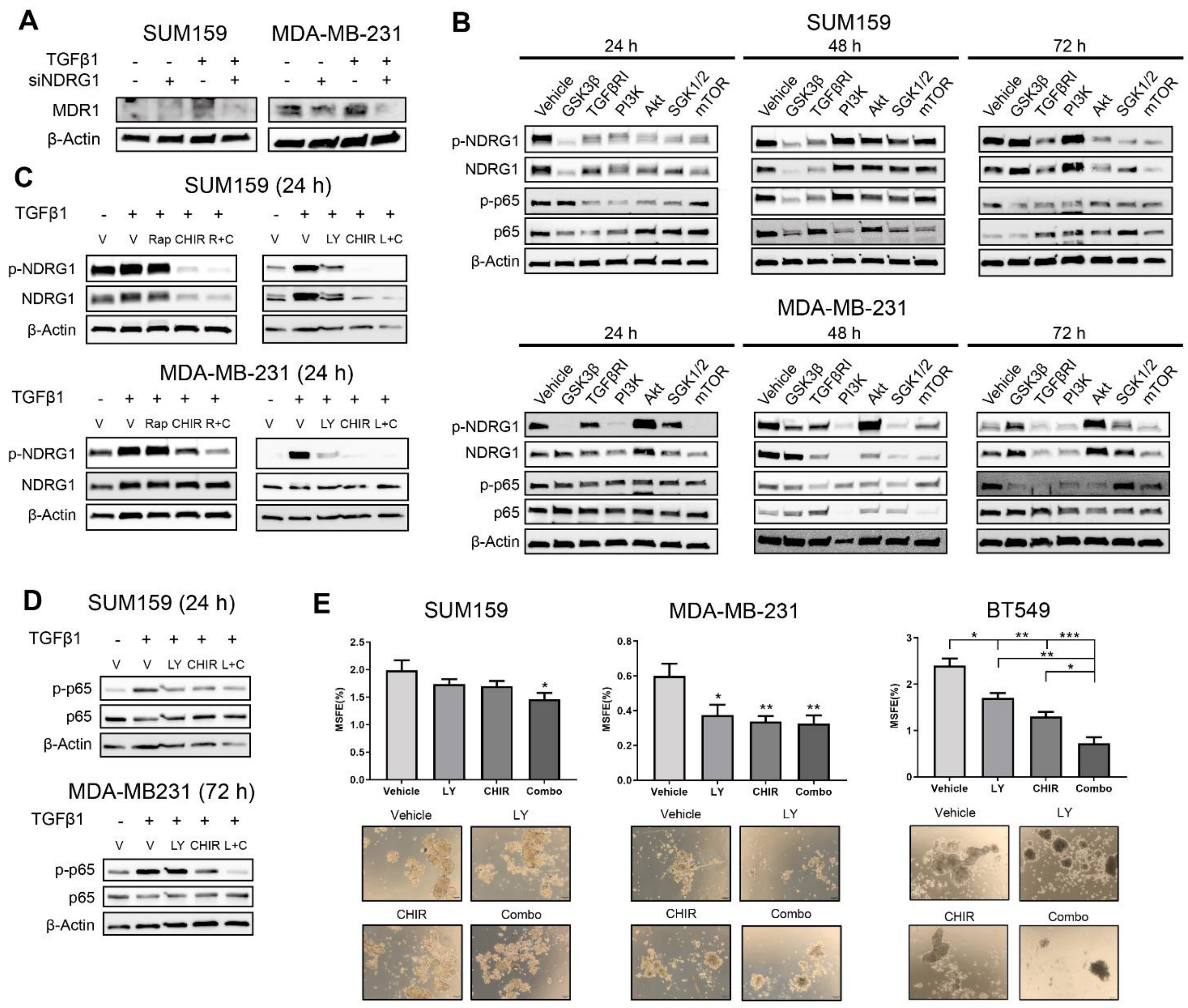

Fig. S5. MDR1 expression, and elucidation of the potential pathway of TGFβ-induced NDRG1 and its targeting. A Western blot of MDR1 in both SUM159 and MDA-MB-231 cells transfected with siNDRG1 treated or not with TGFβ1. **B** Protein expression of NDRG1, p-NDRG1, p-p65, and p65 after the treatment with inhibitors of PI3K, Akt, mTOR, SGK1/2, GSK3β, and TGFβ in SUM159 and MDA-MB-231 cells for 24, 48, and 72h upon TGFβ1 stimulation. **C** Western-blot of NDRG1 and p-NDRG1 after treatment with

rapamycin (Rap), LY2157299 (LY), CHIR99021(CHIR), Rap+CHIR (R+C), Rap+LY (R+L), LY+CHIR (L+C) in SUM159 and MDA-MB-231 cells stimulated with TGFβ1 for 24h. The vehicle is depicted as V. **D** Western-blot of p-p65 and p65 after treatment with LY, CHIR, and LY+CHIR (L+C) in SUM159 and MDA-MB-231 cells, upon stimulation with TGFβ1, for 24 and 72h, respectively, compared with vehicle (V). **E** Primary mammospheres of SUM159, MDA-MB-231, and BT549 cells stimulated or not with TGFβ1 and treated with LY, CHIR, and LY+CHIR (Combo). * $p<0.05$; ** $p<0.01$; *** $p<0.001$.

Supplementary Figure S6

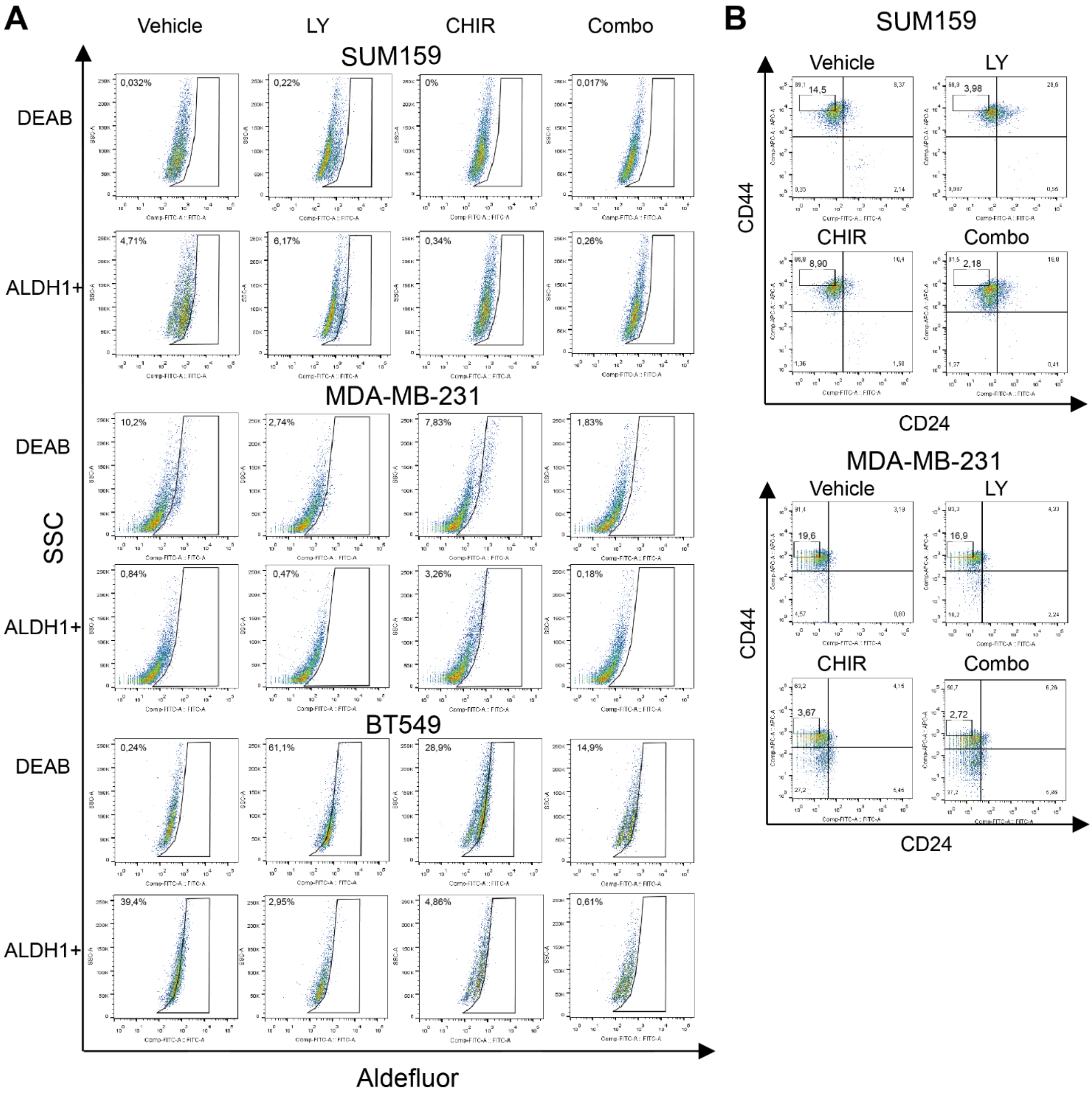

Fig. S6. A Flow cytometric dot plots of aldefluor-positive ($ALDH1^+$) population and DEAB control in secondary mammospheres of SUM159, MDA-MB-231, and BT549 cell lines after treatment with TGFβ1, LY, CHIR, and LY+CHIR (Combo). **B** Flow cytometric dot plots of $CD44^{high}/CD24^-$ in 2MS of SUM159 and MDA-MB-2312 cell lines after treatment with TGFβ1, LY, CHIR, and LY+CHIR (Combo).

Supplementary Tables.

Supplementary Table S1. Univariate analysis of the effect of high/low expression of p-NDRG1 (Thr346) over patient survival.

Variable	Number of patients	% of total	Estimated mean survival (years)	p-value (Log-rank)	HR	Cox regression (95% CI)	p-value
Total p-NDRG1 (Score according to ROC: ≥ 65) (Total n = 83)							
≥ 65	22	26.5	16.82	0.107	1.00	Ref.	0.127
< 65	61	73.5	11.16		0.50	0.2-1.21	
Nuclear p-NDRG1 (Score according to ROC: ≥ 7.5) (Total n = 77)							
≥ 7.5	23	29.9	18.75	0.004*	1.00	Ref.	0.009*
< 7.5	54	70.1	10.08		0.24	0.08-0.7	
Cytoplasmic p-NDRG1 (Score according to ROC: ≥ 12.5) (Total n = 83)							
≥ 12.5	36	43.4	15.45	0.152	1.00	Ref.	0.171
< 12.5	47	56.6	10.97		0.61	0.3-1.23	
Membrane p-NDRG1 (Score according to ROC: ≥ 120) (Total n = 77)							
≥ 120	1	1.3	ND	NA	NA	NA	NA
< 120	76	98.7	13.51				

ND: Not Deceased. NA: Not Available

Supplementary Table S2. Clinical evolution, p-GSK3β (Tyr216) and TGFβ1 staining, and univariate analysis.

**Variable**	**Number of patients**	**% of total**	**Estimated mean survival (years)**	***p*-value (Log-rank)**	***HR***	***Cox regression (95% CI)***	***p-value***
TGFβ1 (total n = 69)							
Positive	57	82.6	13.84	0.146	2.7	0.64-11.5	0.174
Negative	12	17.4	10.99		1.00	Ref.	
Missing	14						
Nuclear							
Positive	6	8.8	10.16	0.218	1.89	0.65-5.52	0.239
Negative	62	91.2	13.08		1.00	Ref.	
Missing	1						
Cytoplasmic							
Positive	57	83.8	13.84	0.197	2.44	0.57-10.34	0.225
Negative	11	16.2	10.79		1.00	Ref.	
Missing	1						
Membrane							
Positive	14	20.6	10.10	0.824	1.10	0.44-2.75	0.829
Negative	54	79.4	14.66		1.00	Ref.	
Missing	1						
p-GSK3β (total n = 72)							
Positive	56	7.8	14.73	0.588	1.00	Ref.	0.597
Negative	16	22.2	11.37		1.26	0.53-2.96	
Nuclear							
Positive	29	40.3	14.25	0.910	1.04	0.49-2.20	0.912
Negative	43	59.7	12.62		1.00	Ref.	
Cytoplasmic							
Positive	49	68.1	15.59	0.128	1.00	Ref.	0.144
Negative	23	31.9	10.47		1.74	0.82-3.69	
Membrane							
Positive	0	0	NA	NA	NA	NA	NA
Negative	72	100	14.379				

NA: Not Available. HR: Hazard Ratio
